# Teixobactin “Swapmers” with l Tail Stereochemistry Retain Antibiotic Activity

**DOI:** 10.1021/acs.joc.4c01674

**Published:** 2024-09-30

**Authors:** James H. Griffin, Ana-Teresa Mendoza, James S. Nowick

**Affiliations:** † Department of Chemistry, University of California—Irvine, Irvine, California 92697, United States; ‡ Department of Pharmaceutical Sciences, University of California—Irvine, Irvine, California 92697, United States

## Abstract

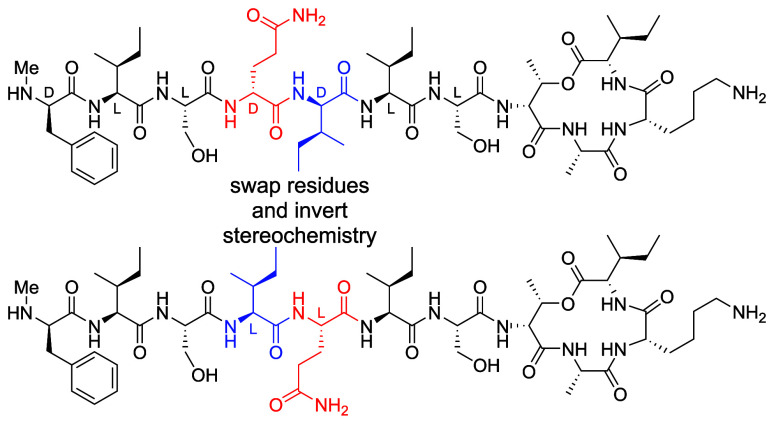

The unusual d-l-l-d-d-l-l pattern of stereochemistry in residues 1–7 of the peptide antibiotic teixobactin is critical to its extraordinary antibiotic activity, creating an unusual amphiphilic β-sheetlike structure that is essential to its mechanism of action. The current study sought to replace the three d-amino acids in the tail with l-amino acids while maintaining amphiphilicity. We find that swapping residues d-Gln_4_ and d-*allo*-Ile_5_ in *O*-acyl isopeptide prodrugs of teixobactin permits the introduction of l-stereochemistry with retention of antibiotic activity. Nevertheless, modifying the *N*-terminal stereochemistry results in a loss of antibiotic activity.

The peptide antibiotic teixobactin achieves its remarkable activity through the unique pattern of d-l-l-d-d-l-l stereochemistry in amino acid residues 1–7.^1−3^ Teixobactin comprises a macrolactone ring (residues 8–11) and a linear tail (residues 1–7); the ring binds the pyrophosphate anion of lipid II and related bacterial cell wall precursor molecules, and the tail forms an amphiphilic β-strand that induces β-sheet assembly (Figure 1). The pattern of hydrophobic and hydrophilic amino acid side chains works in concert with the alternating d and l backbone stereochemistry to give rise to the amphiphilic β-strand, and the multiple interactions conferred by this supramolecular assembly contribute to high antibiotic activity.

**Figure 1:**
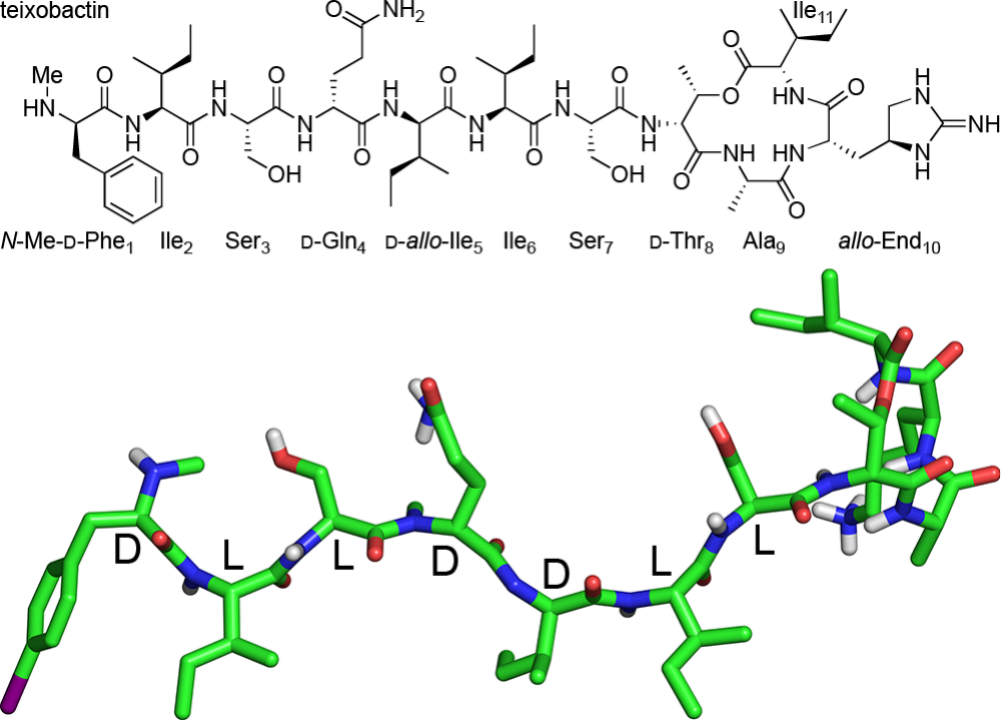
Teixobactin structure and X-ray crystallographic structure of a teixobactin analogue (*N*-Me-d-Phe^I^_1_,*N*-Me-d-Gln_4_,Lys_10_-teixobactin, PDB entry 6E00(2)). The stereochemistry in each amino acid residue 1–7 is indicated.

Previous structure–activity relationship studies have shown that teixobactin does not tolerate stereochemical modifications to the linear tail. Mutating *N*-Me-d-Phe_1_, d-Gln_4_, or d-*allo*-Ile_5_ to their respective l-amino acids results in a loss of antibiotic activity.^4−7^ The stereochemistry of these residues is critical because they enable the tail to adopt an unusual β-strandlike conformation. In a β-strand with all-l stereochemistry, the side chains are arranged down–up–down–up–down–up–down (Figure 2A). In teixobactin, the stereochemical pattern is instead down–down–up–up–down–down–up (Figure 2B). This pattern, coupled with the arrangement of hydrophobic and hydrophilic amino acids, gives rise to the amphiphilicity of the teixobactin tail.

**Figure 2:**
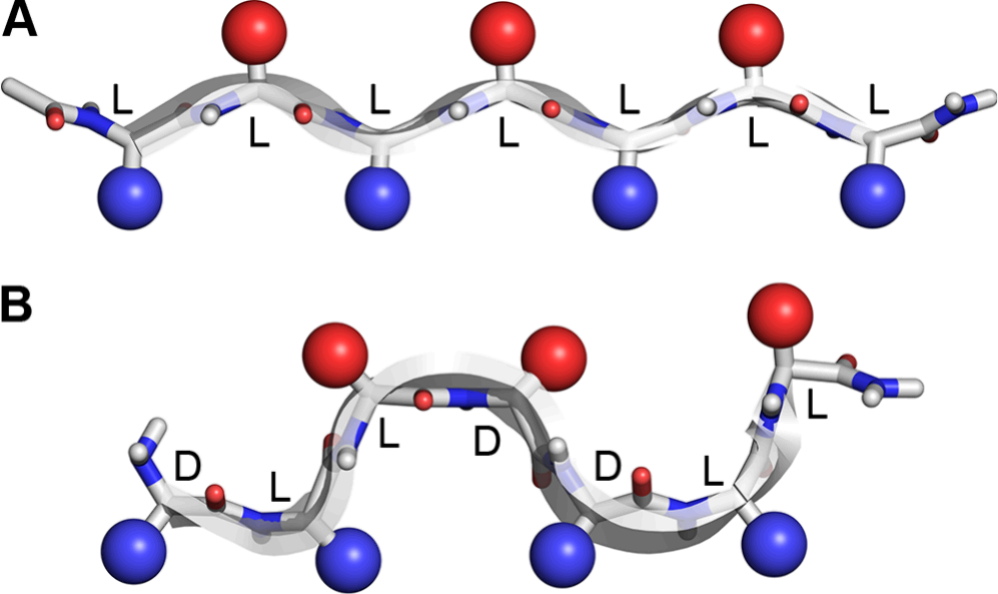
Cartoon representations of (A) an l-amino acid β-strand and (B) a teixobactin β-strand. Polar and nonpolar residues are shown as red and blue spheres, respectively. The cartoon representation in A is an idealized β-strand, and the cartoon representation in B is adapted from PDB 6PSL.^8^

In the current paper, we ask whether we can alter the backbone stereochemistry of teixobactin while retaining antibiotic activity by concurrently altering both the stereochemistry and the pattern of hydrophobic and hydrophilic residues of its linear tail. Of the three d-amino acids in the tail, d-Gln_4_ and d-*allo*-Ile_5_ are adjacent to each other and have opposite philicity. We hypothesized that pairwise swapping of d-Gln_4_ and d-*allo*-Ile_5_ to l-Ile_4_ and l-Gln_5_ would preserve the amphiphilicity of the resulting β-strand. The resulting “swapmer” has l stereochemistry in each tail residue except the *N*-terminal *N*-Me-d-Phe_1_. We tested this hypothesis with analogues of teixobactin containing lysine or arginine at position 10, because the native l-*allo*-enduracidine (l-*allo*-End) amino acid is not commercially available (Figure 3).

**Figure 3:**
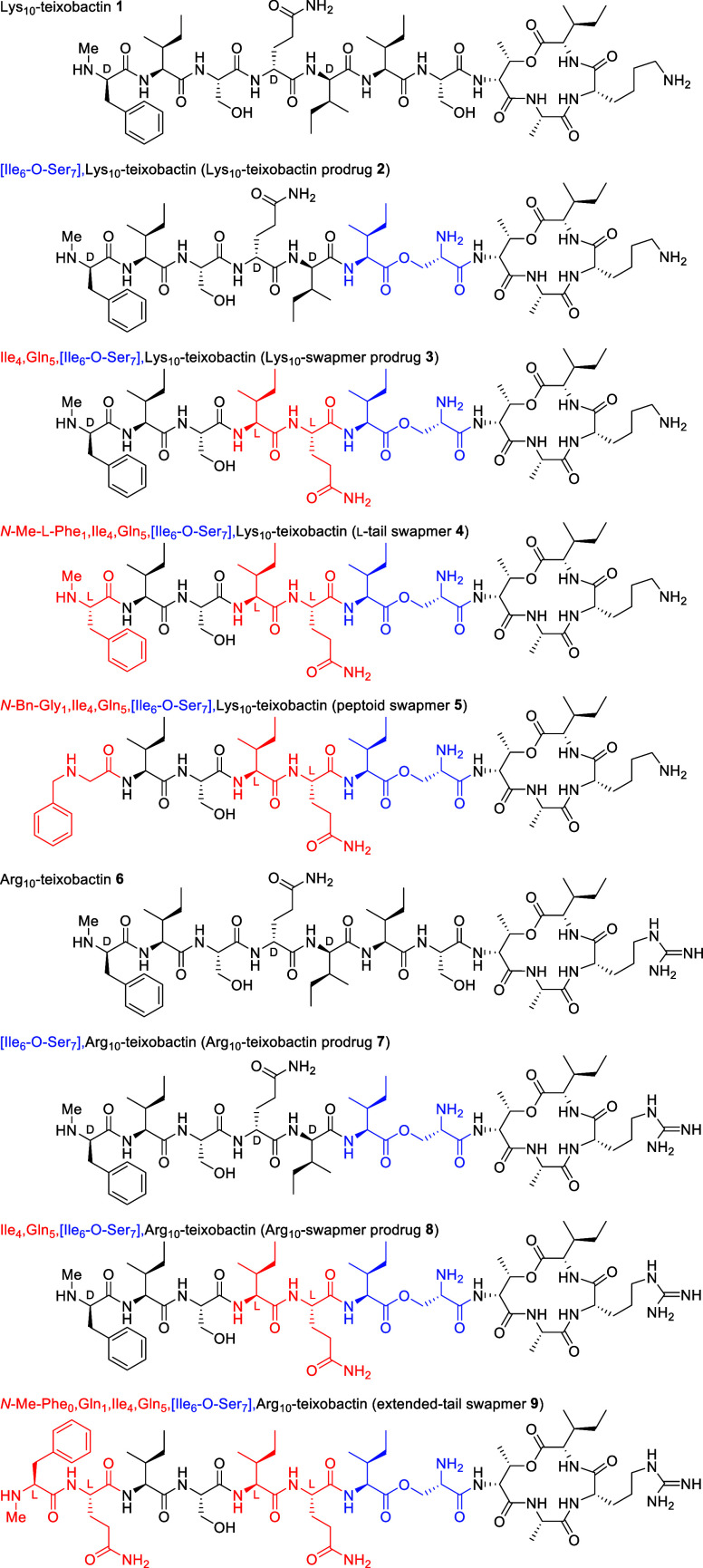
Chemical structures of teixobactin analogues prepared and studied. Amino acid stereochemistry is indicated for residues 1, 4, and 5. Stereochemically modified residues are highlighted in red. *O*-Acyl dipeptide units are highlighted in blue. We studied prodrugs containing *O*-acyl dipeptide units to overcome problems with solubility.

Although our laboratory has synthesized more than 200 teixobactin derivatives, we were unable to synthesize the swapmer Ile_4_,Gln_5_,Lys_10_-teixobactin. In monitoring the solid-phase coupling reactions by LC-MS and analytical HPLC, we observed poor coupling efficiencies, which are hallmarks of aggregation-prone peptides. To address this problem, we employed a strategy that we have previously used for aggregation-prone peptides: the incorporation of an *O*-acyl linkage to serine.^9,10^ This “isoacyl dipeptide” strategy was first introduced by Kiso and co-workers to facilitate the preparation of aggregation-prone peptides.^11^ The resulting isopeptides then convert cleanly and rapidly at neutral pH to the corresponding peptides.^12^ We have previously used this strategy to create prodrugs of teixobactin analogues, which convert to the corresponding teixobactin analogues during the conditions of minimum inhibitory concentration (MIC) assays.^9,10^ These prodrugs exhibit comparable or better antibiotic activity in the MIC assays. We used this prodrug strategy to prepare, purify, and study Ile_4_,Gln_5_,[Ile_6_-O-Ser_7_],Lys_10_-teixobactin and Ile_4_,Gln_5_,[Ile_6_-O-Ser_7_],Arg_10_-teixobactin. We refer to these compounds as Lys_10_-swapmer prodrug **3** and Arg_10_-swapmer prodrug **8**, respectively (Figure 3).

We determined the MIC values of these teixobactin swapmer prodrugs against the Gram-positive bacteria *Bacillus subtilis*, *Staphylococcus epidermidis*, methicillin-susceptible *Staphylococcus aureus* (MSSA), and methicillin-resistant *Staphylococcus aureus* (MRSA) in a broth microdilution assay as described previously.^9,10^ We used the antibiotic vancomycin as a positive control and the Gram-negative bacterium *Escherichia coli* as a negative control. The MIC values for Lys_10_-teixobactin **1**, Lys_10_-teixobactin prodrug **2**, Arg_10_-teixobactin **6**, and Arg_10_-teixobactin prodrug **7** are shown in Table 1 for comparison with the swapmer analogues.

**Table 1:** MIC Values of Teixobactin, Teixobactin Prodrugs, and “Swapmer” Analogues in μg/mL^a^

	*Bacillus subtilis* ATCC 6051	*Staphylococcus epidermidis* ATCC 14990	*Staphylococcus aureus* (MSSA) ATCC 29213	*Staphylococcus aureus* (MRSA) ATCC 700698	*Escherichia coli* ATCC 10798
Lys_10_-teixobactin (**1**)	≤0.0313	1	2	2	>32
[Ile_6_-O-Ser_7_],Lys_10_-teixobactin (Lys_10_-teixobactin prodrug **2**)	≤0.0313	1	2	1–2	>32
Ile_4_,Gln_5_,[Ile_6_-O-Ser_7_],Lys_10_-teixobactin (Lys_10_-swapmer prodrug **3**)	4	4	8	8	>32
*N*-Me-l-Phe_1_,Ile_4_,Gln_5_,[Ile_6_-O-Ser_7_],Lys_10_-teixobactin (l-tail swapmer **4**)	≥32	>32	>32	>32	>32
*N*-Bn-Gly_1_,Ile_4_,Gln_5_,[Ile_6_-O-Ser_7_],Lys_10_-teixobactin (peptoid swapmer **5**)	32	>32	>32	>32	>32
Arg_10_-teixobactin (**6**)	≤0.0313	0.5	2	2	>32
[Ile_6_-O-Ser_7_],Arg_10_-teixobactin (Arg_10_-teixobactin prodrug **7**)	0.0625	0.5	2	1	>32
Ile_4_,Gln_5_,[Ile_6_-O-Ser_7_],Arg_10_-teixobactin (Arg_10_-swapmer prodrug **8**)	2	2	4	4	>32
*N*-Me-Phe_0_,Gln_1_,Ile_4_,Gln_5_,[Ile_6_-O-Ser_7_],Arg_10_-teixobactin (extended-tail swapmer **9**)	>32	>32	>32	>32	>32
vancomycin	0.125–0.25	1–2	1–2	4	>32

a MIC assays were performed in the presence of 0.002% polysorbate 80.

Lys_10_-swapmer prodrug **3** has MIC values between 4–8 μg/mL against the Gram-positive bacteria tested, and Arg_10_-swapmer prodrug **8** has MIC values between 2–4 μg/mL against the Gram-positive bacteria tested (Table 1). Compared to the unswapped analogues **1**, **2**, **6**, and **7**, swapmers **3** and **8** have 2–4-fold reduced activity against *S. epidermidis*, MSSA, and MRSA. The activities of Lys_10_-swapmer prodrug **3** and Arg_10_-swapmer prodrug **8** support the hypothesis that pairwise swapping of residues 4 and 5 with inversion of stereochemistry in teixobactin preserves antibiotic activity. However, the reduction in the activity of these swapmers shows that there is still some loss of activity associated with the incorporation of l amino acids at these positions. *B. subtilis* is exceptionally sensitive to unswapped teixobactin analogues **1**, **2**, **6**, and **7**, with MICs of ≤0.0625 μg/mL. Although swapmers **3** and **8** do not reflect this extraordinary activity, their activities against *B. subtilis* are comparable to their activities against the other Gram-positive bacteria.

To test whether a teixobactin analogue with all-l tail stereochemistry would retain antibiotic activity, we prepared *N*-Me-l-Phe_1_,Ile_4_,Gln_5_,[Ile_6_-O-Ser_7_],Lys_10_-teixobactin (l-tail swapmer **4**), in which the only remaining d-amino acid in the tail, *N*-Me-d-Phe_1_, is instead l. While this design does not preserve amphiphilicity in the idealized β-strand, we envisioned that the flexibility of the *N*-terminal residue might still allow the peptide to adopt an amphiphilic conformation. Nevertheless, the l-tail swapmer **4** is virtually inactive against all bacteria in the concentration range tested. We next hypothesized that removal of stereochemistry at position 1 would allow activity in an analogue with an otherwise all-l tail, and subsequently prepared *N*-Bn-Gly_1_,Ile_4_,Gln_5_,[Ile_6_-O-Ser_7_],Lys_10_-teixobactin (peptoid swapmer **5**), in which the *N*-terminal residue is achiral. Peptoid swapmer **5** is only very weakly active against *B. subtilis* and is inactive against all other bacteria tested. These two results indicate that even in a swapmer analogue with otherwise all-l tail stereochemistry, d stereochemistry at position 1 is necessary for antibiotic activity. Previous structure–activity relationship studies of teixobactin by Albericio, Li, and our group have shown that residue 1 does not tolerate modifications, including alanine or stereochemical mutation.^5,7,13−15^

In a third and final attempt to introduce l-stereochemistry at the *N*-terminus of teixobactin analogues, we prepared a tail-extended analogue. To preserve an *N*-terminal phenylalanine side chain in an idealized, all-l, amphiphilic β-strand, we prepared *N*-Me-Phe_0_,Gln_1_,Ile_4_,Gln_5_,[Ile_6_-O-Ser_7_],Arg_10_-teixobactin (extended-tail swapmer **9**), in which the linear tail is extended by one amino acid residue. We incorporated l-glutamine at position 1 to duplicate the pattern of residues 4 and 5 and incorporated *N*-Me-l-phenylalanine at “position 0” to match the *N*-terminal residue of teixobactin. As with our two preceding attempts to eliminate the d-stereochemistry at position 1, extended-tail swapmer **9** was completely inactive against all bacteria tested. This result indicates that we cannot create an active all-l swapmer in which the *N*-terminal *N*-Me-d-phenylalanine is replaced with an amphiphilic l–l dipeptide unit, and further cements that the *N*-Me-d-Phe_1_ residue is critical to the antibiotic activity of teixobactin.

The chirality of the peptide tail of teixobactin is remarkably important for its activity. Although stereochemical mutation of individual residues abrogates the activity of teixobactin analogues, pairwise swapping of residues 4 and 5 with inversion of stereochemistry does not (Figure 4). This pairwise swapping of d-Gln_4_ and d-*allo*-Ile_5_ to l-Ile_4_ and l-Gln_5_ allows for retention of the critical amphiphilic structure and results in only a 2–4-fold loss in activity. Nevertheless, further efforts to alter the stereochemistry of the tail—specifically, the *N*-terminal *N*-Me-d-phenylalanine residue—did not result in active analogues. We envision that our exploration of these principles of amphiphilicity in the tail of teixobactin could enable the development of new antibiotics consisting of (1) an all-l or all-d amphiphilic tail that self-assembles through β-sheet interactions and (2) a macrocycle that targets the pyrophosphate group of lipid II and related bacterial cell wall precursors.

**Figure 4:**
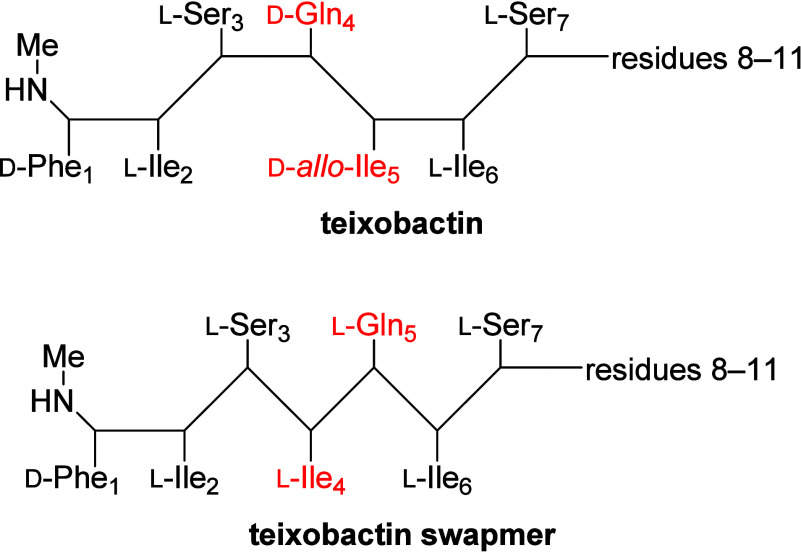
Schematic diagram of teixobactin swapmers illustrating the stereochemical effect of the d-Gln_4_,d-*allo*-Ile_5_ to l-Ile_4_,l-Gln_5_ swap.

## Experimental Section

### General Information

Methylene chloride (CH_2_Cl_2_) was passed through alumina under argon prior to use. Amine-free *N*,*N*-dimethylformamide (DMF) was purchased from Alfa Aesar. Fmoc-d-*allo*-Ile-OH was purchased from Santa Cruz Biotechnology. Boc-Ser(Fmoc-Ile)-OH was purchased from AAPPTec. Fmoc-*N*-Bn-Gly-OH and other protected amino acids were purchased from Chem-Impex. Vancomycin was purchased from Sigma-Aldrich. All peptides were prepared and studied as trifluoroacetate salts. Peptides were first purified on a Biotage Isolera One flash column chromatography instrument equipped with a Biotage SfarBio C18 D Duo 300 Å 20 μm 25 g column. Peptides were then further purified by preparative reversed-phase HPLC on a Shimadzu instrument equipped with an Agilent Zorbax 7 μm 300SB-C18 column (250 mm × 21.2 mm). Analytical reversed-phase HPLC was performed on an Agilent 1260 Infinity II instrument equipped with a Phenomonex bioZen PEPTIDE 2.6 μm XB-C18 column (150 mm × 4.6 mm). LC-MS analysis was performed using a Waters Acuity QDa UPLC/MS. HPLC-grade acetonitrile (MeCN) and deionized water (18 MΩ) containing 0.1% trifluoroacetic acid (TFA) were used as solvents for both preparative and analytical reversed-phase HPLC. Deionized water (18 MΩ) was obtained from a ThermoScientific Barnstead GenPure Pro water purification system. Glass solid-phase peptide synthesis vessels with fritted disks and BioRad Polyprep columns were used for the solid-phase peptide synthesis. Bacteria were incubated in a Thermo Fisher Scientific MaxQ Shaker 6000.

### Peptide Synthesis of Teixobactin Analogues

We synthesized teixobactin analogues as the trifluoroacetate (TFA) salt by manual solid-phase peptide synthesis of the corresponding linear peptide on 2-chlorotrityl resin, followed by on-resin esterification, solution-phase cyclization, deprotection, and purification as previously described.^9^ A step-by-step procedure is detailed below.

#### Resin Loading

2-Chlorotrityl chloride resin (300 mg, 1.07 mmol/g, 0.32 mmol total) was swelled with dry CH_2_Cl_2_ (8 mL) in a Bio-Rad Poly-Prep chromatography column (10 mL) for 30 min with gentle rocking. The CH_2_Cl_2_ was drained from resin, a solution of Fmoc-Lys(Boc)-OH (150 mg, 0.32 mmol) or Fmoc-Arg(Pbf)-OH (208 mg, 0.32 mmol) in 2,4,6-collidine (300 μL) and CH_2_Cl_2_ (8 mL) was added, and the suspension was gently rocked for 12–16 h. The solution was drained from resin; a mixture of CH_2_Cl_2_/CH_3_OH/*N*,*N*-diisopropylethylamine (DIPEA) (8.5:1:0.5, 10 mL) was immediately added, and the resin was gently rocked for 1 h to cap unreacted 2-chlorotrityl resin sites. The solution was drained from the resin, and the resin was washed three times with CH_2_Cl_2_. Resin loading was quantified as previously described.^16^ Briefly, ca. 1.0 mg of dried, loaded resin was transferred to a scintillation vial containing 3.0 mL of 20% (v/v) piperidine/DMF and gently shaken for 10 min. The absorbance of the solution was measured at 290 nm to determine the concentration of the piperidine-dibenzofulvene (Fmoc) adduct, which is proportional to the amount of loaded amino acid. Typical resin loadings of 0.43–0.56 mmol/g were observed for lysine, and typical resin loadings of 0.11–0.16 mmol/g were observed for arginine.

#### Linear Peptide Synthesis

The loaded resin was suspended in dry DMF and transferred to a solid-phase peptide synthesis vessel. For peptides **1** and **6**, residues 9 through 1 were manually coupled using Fmoc-Ala-OH, Fmoc-d-Thr-OH, Fmoc-Ser(*t*-Bu)-OH, Fmoc-Ile-OH, Fmoc-d-*allo*-Ile-OH, Fmoc-d-Gln(Trt)-OH, Fmoc-Ser(*t*-Bu)-OH, Fmoc-Ile-OH, and Boc-*N*-Me-d-Phe-OH. For residue 8, side-chain unprotected Fmoc-d-Thr-OH was used to enable the later esterification step. For the *N*-terminal residue, Boc-*N*-Me-d-Phe-OH was used to prevent cross-reactivity during the cyclization step. For prodrug peptides **2**–**5** and **7**–**9**, Boc-Ser(Fmoc-Ile)-OH was used in place of Fmoc-Ser(*t*-Bu)-OH and Fmoc-Ile-OH when coupling residues 7 and 6. For *N*-terminally modified peptides **4**, **5**, and **9**, Boc-*N*-Me-l-Phe-OH or Boc-*N*-Bn-Gly-OH was used in place of Boc-*N*-Me-d-Phe-OH. Amino acids were coupled through the following cycles: *i.* Fmoc-deprotection with 20% (v/v) piperidine in DMF (5 mL) for 20 min, *ii.* washing with DMF (3 × 5 mL), *iii.* coupling of the amino acid (4 equiv) with HCTU (4 equiv) in 20% (v/v) 2,4,6-collidine in DMF (5 mL) for 30 min (60 min for l-to-d or d-to-l couplings, or when coupling to β-branched amino acids), and *iv.* washing with DMF (3 × 5 mL).

#### Ile_11_ Esterification

The resin was drained, washed with CH_2_Cl_2_ (3 × 5 mL), and transferred to a clean Bio-Rad Poly-Prep chromatography column. In a test tube, Fmoc-Ile-OH (10 equiv) and diisopropylcarbodiimide (10 equiv) were dissolved in CH_2_Cl_2_ (5 mL). The resulting solution was filtered through a 0.20-μm nylon filter, and 4-dimethylaminopyridine (1 equiv) was added to the filtrate. The resulting solution was transferred to the resin and gently rocked for 1 h. The solution was drained and then washed with CH_2_Cl_2_ (3 × 5 mL) and DMF (3 × 5 mL).

#### Fmoc Deprotection of Ile_11_ and Cleavage of the Linear Peptide from Chlorotrityl Resin

The Fmoc protecting group on Ile_11_ was removed by adding 20% (v/v) piperidine in DMF and gently rocking for 30 min. The solution was drained and then washed with DMF (3 × 5 mL) and CH_2_Cl_2_ (3 × 5 mL). The linear peptide was cleaved from the resin by rocking the resin in a solution of 20% (v/v) 1,1,1,3,3,3-hexafluoroisopropanol (HFIP) in CH_2_Cl_2_ (10 mL) for 1 h. Upon addition of the HFIP solution, the resin beads and solution change color from yellow and clear, respectively, to red. The suspension was filtered, and the filtrate was collected in a 250 mL round-bottomed flask. The resin was washed with an additional HFIP solution (10 mL) for 30 min and then filtered into the same flask. The combined filtrates were concentrated by rotary evaporation and further dried by a vacuum pump to afford the crude protected linear peptide, which was cyclized without further purification.

#### Cyclization of the Linear Peptide

The crude protected linear peptide was dissolved in dry DMF (125 mL). HOAt (6 equiv) and HATU (6 equiv) were dissolved in DMF (8 mL) in a test tube. The HOAt/HATU solution was added to the flask containing the dissolved peptide, and the mixture was stirred under nitrogen for 30 min. Diisopropylethylamine (300 μL) was added to the flask, and the mixture was stirred under nitrogen for an additional 16–20 h. The reaction mixture was concentrated by rotary evaporation and further dried by a vacuum pump to afford the crude protected cyclized peptide, which was immediately subjected to global deprotection.

#### Global Deprotection and Ether Precipitation

The protected cyclic peptide was dissolved in TFA:triisopropylsilane (TIPS):H_2_O (9:0.5:0.5, 10 mL) in a 250 mL round-bottomed flask equipped with a stir bar, and the solution was stirred under nitrogen for 1 h. During the 1 h deprotection, two 50 mL conical tubes containing 40 mL each of dry Et_2_O were chilled on ice. After the 1 h deprotection, the peptide solution was split between the two conical tubes of chilled Et_2_O. The tubes were then centrifuged at 600 × *g* for 10 min and decanted. The pelleted peptides were dried under nitrogen.

#### Reversed-Phase HPLC Purifications

The peptide was dissolved in 20% (v/v) CH_3_CN in H_2_O (5 mL) containing 0.1% TFA, injected on the Biotage instrument (General Information) at 20% CH_3_CN, and eluted with a gradient of 20%–40% CH_3_CN over 15 min. The fractions containing the desired peptide as determined by LC-MS were concentrated by rotary evaporation, diluted in 20% (v/v) CH_3_CN in H_2_O (5 mL) containing 0.1% TFA, injected on the Shimadzu instrument (General Information) at 20% CH_3_CN, and eluted with a gradient of 20%–40% CH_3_CN over 80 min. The collected fractions were analyzed by analytical HPLC and LC-MS, and the pure fractions were concentrated by rotary evaporation and lyophilized. Note that Fmoc-Ile_11_-OH typically undergoes ca. 30% epimerization during coupling to the OH group of d-Thr_8_.^17^ The epimeric peptide product is removed during the HPLC purification.

### Minimum Inhibitory Concentration (MIC) Assays

We performed minimum inhibitory concentration (MIC) assays as previously described.^9^ A concise procedure is detailed below.

#### Preparing Peptide Stock Solutions

Stock solutions of teixobactin analogues were prepared gravimetrically by dissolving an appropriate amount of peptide in 20 mg/mL sterile DMSO. The stock solutions were stored at −20 °C for subsequent experiments.

#### Preparing Bacterial Cultures

*Bacillus subtilis* (ATCC 6051), *Staphylococcus epidermidis* (ATCC 14990), methicillin-susceptible *Staphylococcus aureus* (MSSA) (ATCC 29213), and *Escherichia coli* (ATCC 10798) were cultured from glycerol stocks in Mueller-Hinton broth containing 0.002% polysorbate 80 overnight in a shaking incubator at 37 °C. Methicillin-resistant *Staphylococcus aureus* (MRSA) (ATCC 700698) was cultured from a glycerol stock in brain hearth infusion broth containing 0.002% polysorbate 80 overnight in a shaking incubator at 37 °C. After overnight incubation, each culture was diluted with the appropriate media (containing 0.002% polysorbate 80) to 1 × 10^6^ colony-forming units (CFU)/mL as previously described.^12,15,16^ Briefly, we have previously determined the CFU/mL for each bacterium at OD_600_ = 0.075 for 200 μL of bacterial culture in a 96-well plate. The OD_600_ of each overnight culture was determined, and then each culture was diluted to OD_600_ = 0.075. Cultures were further diluted to achieve 1 × 10^6^ CFU/mL solutions.

#### 96-Well Plate Setup

Aliquots of the 20 mg/mL peptide stock solutions were diluted to make a 64 μg/mL solution in Mueller-Hinton broth containing 0.002% polysorbate 80 and a 64 μg/mL solution in brain heart infusion broth containing 0.002% polysorbate 80. The solutions were mixed by pipetting to ensure homogeneity. A 200-μL aliquot of a 64 μg/mL solution was transferred to a 96-well plate. 2-fold serial dilutions were made with media containing 0.002% polysorbate 80 across the 96-well plate to achieve a final volume of 100 μL in each well (initial concentrations ranging from 64 to 0.0625 μg/mL). A 100-μL aliquot of a 1 × 10^6^ CFU/mL bacterial solution was added to each well in the series, resulting in final bacterial concentrations of 5 × 10^5^ CFU/mL in each well. As 100 μL of bacteria were added to each well, the concentration of peptide was also diluted 2-fold (final concentrations ranging from 32 μg/mL to 0.03125 μg/mL). Each plate was covered with a lid and incubated at 37 °C for 16 h. The optical density measurements were measured at 600 nm using a 96-well UV–vis plate reader (MultiSkan GO, Thermo Scientific). The MIC values were taken as the lowest concentration that had no bacterial growth. MIC assays for each compound were performed in triplicate to ensure reproducibility.

### HPLC Conditions and MS Results

Analytical RP-HPLC was performed on a C18 column with an elution gradient of 5–100% CH_3_CN + 0.1% TFA over 20 min.

#### Lys_10_-teixobactin (**1**)

MS (ESI) *m*/*z*: [M + H]^+^ Calcd for C_58_H_98_N_13_O_15_ 1216.7300; Found 1216.7250.

#### [Ile_6_-O-Ser_7_],Lys_10_-teixobactin (**2**)

MS (ESI) *m*/*z*: [M + H]^+^ Calcd for C_58_H_98_N_13_O_15_ 1216.7300; Found 1216.7225.

#### Ile_4_,Gln_5_,[Ile_6_-O-Ser_7_],Lys_10_-teixobactin (**3**)

MS (ESI) *m*/*z*: [M + H]^+^ Calcd for C_58_H_98_N_13_O_15_ 1216.7300; Found 1216.7242.

#### N-Me-l-Phe_1_,Ile_4_,Gln_5_,[Ile_6_-O-Ser_7_],Lys_10_-teixobactin (**4**)

MS (ESI) *m*/*z*: [M + H]^+^ Calcd for C_58_H_98_N_13_O_15_ 1216.7300; Found 1216.7175.

#### N-Bn-Gly_1_,Ile_4_,Gln_5_,[Ile_6_-O-Ser_7_],Lys_10_-teixobactin (**5**)

MS (ESI) *m*/*z*: [M + H]^+^ Calcd for C_57_H_96_N_13_O_15_ 1202.7143; Found 1202.7050.

#### Arg_10_-teixobactin (**6**)

MS (ESI) *m*/*z*: [M + H]^+^ Calcd for C_58_H_98_N_15_O_15_ 1244.7361; Found 1244.7481.

#### [Ile_6_-O-Ser_7_],Arg_10_-teixobactin (**7**)

MS (ESI) *m*/*z*: [M + H]^+^ Calcd for C_58_H_98_N_15_O_15_ 1244.7361; Found 1244.7464.

#### Ile_4_,Gln_5_,[Ile_6_-O-Ser_7_],Arg_10_-teixobactin (**8**)

MS (ESI) *m*/*z*: [M + H]^+^ Calcd for C_58_H_98_N_15_O_15_ 1244.7361; Found 1244.7318.

#### N-Me-Phe_0_,Gln_1_,Ile_4_,Gln_5_,[Ile_6_-O-Ser_7_],Arg_10_-teixobactin (**9**)

MS (ESI) *m*/*z*: [M + H]^+^ Calcd for C_63_H_106_N_17_O_17_ 1372.7947; Found 1372.7905.

## Data Availability

The data underlying this study are available in the published article and its Supporting Information.

## Supporting Information Available

HPLC and MS characterization data (PDF)
