# Supplementary material for: Teixobactin “Swapmers” with l Tail Stereochemistry Retain Antibiotic Activity
Source: J Org Chem. 2024 Sep 30;89(20):15325–30. doi: 10.1021/acs.joc.4c01674 (PMC11494653; doi:10.1021/acs.joc.4c01674)

Supporting information for:
Teixobactin "Swapmers" With L Tail Stereochemistry Retain Antibiotic Activity

James H. Griffin,[a] Ana-Teresa Mendoza,[a] and James S. Nowick[a,b,*]

[a] Department of Chemistry, University of California Irvine, Irvine, CA 92697.
[b] Department of Pharmaceutical Sciences, University of California Irvine, Irvine, CA 92697.

Table of Contents

Figure S1 – Synthesis of Ile_4,Gln_5,[Ile_6-O-Ser_7],Lys_{10}-teixobactin **3** **S2**

Peptide Characterization Data

Lys_{10}-teixobactin **1** **S3**
Analytical HPLC trace S3
Mass spectrum S4

[Ile_6-O-Ser_7],Lys_{10}-teixobactin **2** **S5**
Analytical HPLC trace S5
Mass spectrum S6

Ile_4,Gln_5,[Ile_6-O-Ser_7],Lys_{10}-teixobactin **3** **S7**
Analytical HPLC trace S7
Mass spectrum S8

N-Me-L-Phe_1,Ile_4,Gln_5,[Ile_6-O-Ser_7],Lys_{10}-teixobactin **4** **S9**
Analytical HPLC trace S9
Mass spectrum S10

N-Bn-Gly_1,Ile_4,Gln_5,[Ile_6-O-Ser_7],Lys_{10}-teixobactin **5** **S11**
Analytical HPLC trace S11
Mass spectrum S12

Arg_{10}-teixobactin **6** **S13**
Analytical HPLC trace S13
Mass spectrum S14

[Ile_6-O-Ser_7],Arg_{10}-teixobactin **7** **S15**
Analytical HPLC trace S15
Mass spectrum S16

Ile_4,Gln_5,[Ile_6-O-Ser_7],Arg_{10}-teixobactin **8** **S17**
Analytical HPLC trace S17
Mass spectrum S18

N-Me-Phe_0,Gln_1,Ile_4,Gln_5,[Ile_6-O-Ser_7],Arg_{10}-teixobactin **9** **S19**
Analytical HPLC trace S19
Mass spectrum S20

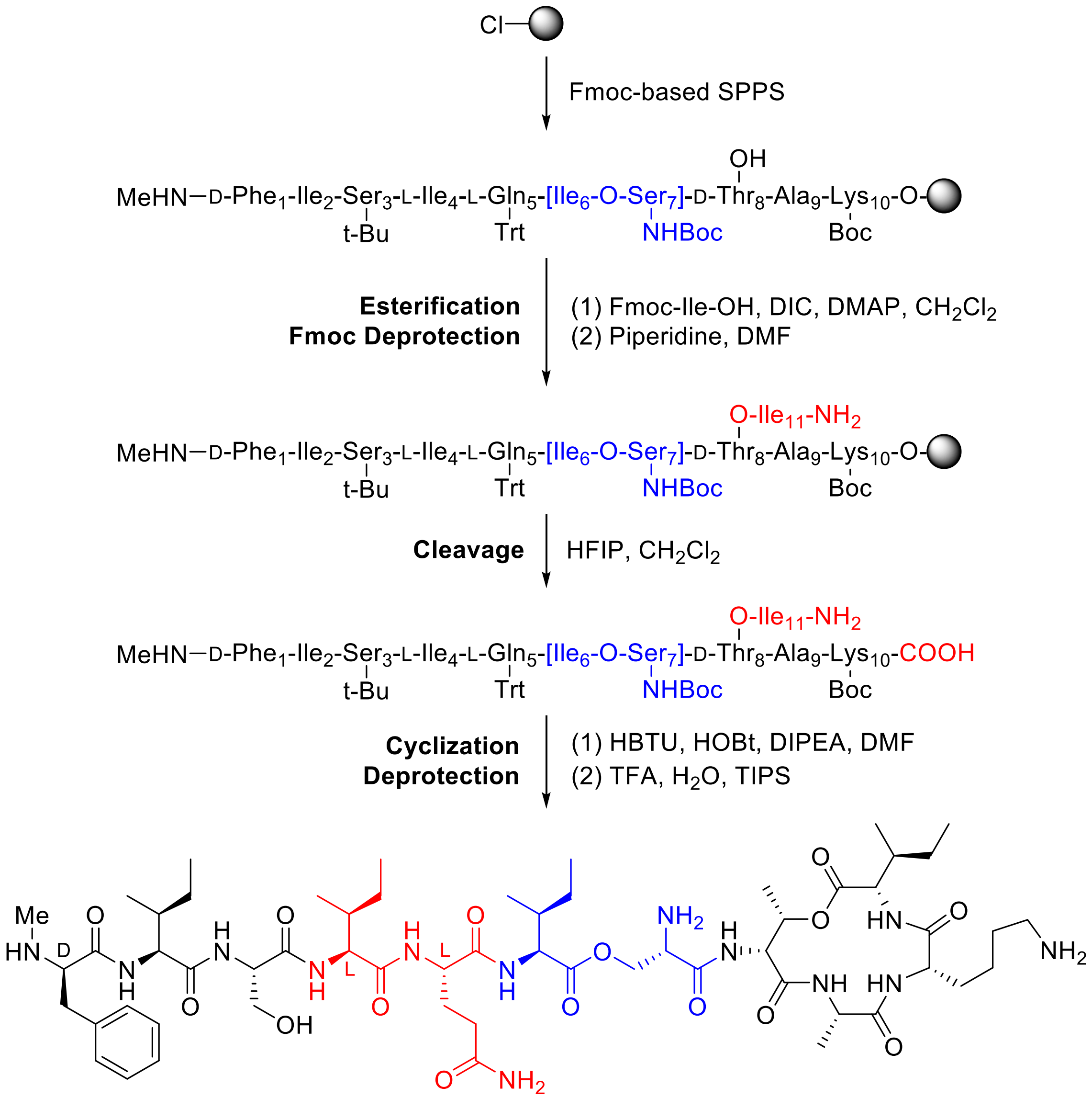

Ile$_4$,Gln$_5$,[Ile$_6$-O-Ser$_7$],Lys$_{10}$-teixobactin (Lys$_{10}$-swapmer prodrug **3**)

Figure S1. Synthesis of Ile$_4$,Gln$_5$,[Ile$_6$-O-Ser$_7$],Lys$_{10}$-teixobactin **3**.

Peptide Characterization Data

Solvent blank

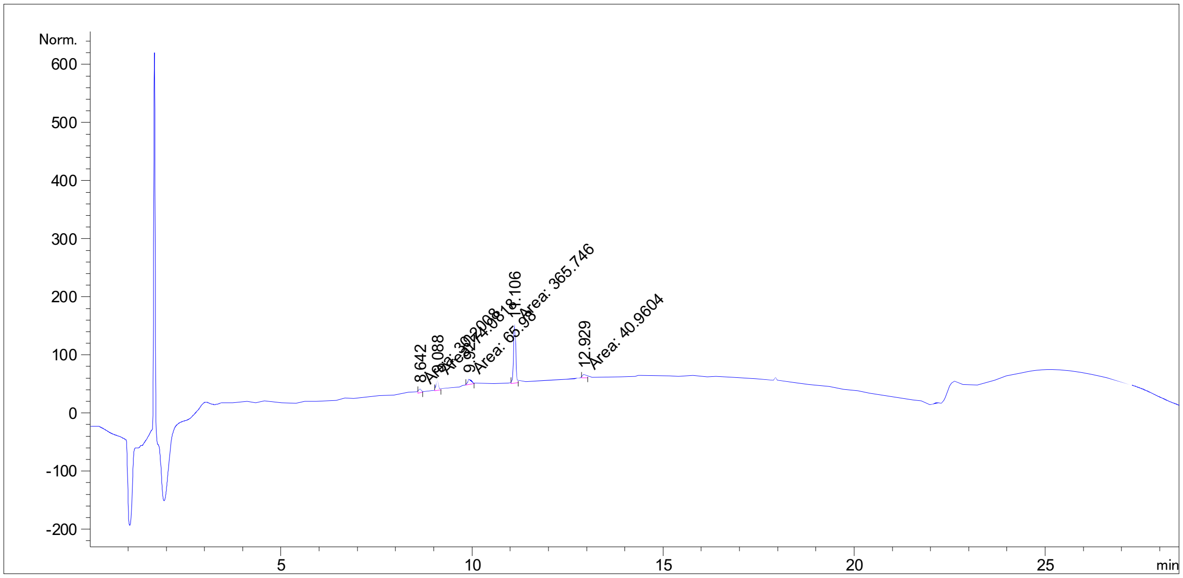

Peak #	RetTime [min]	Type	Width [min]	Area [mAU*s]	Height [mAU]	Area %
1	8.642	MM	0.0629	30.20078	8.00599	5.234 4
2	9.088	MM	0.0628	74.08182	19.65947	12.839 8
3	9.922	MM	0.0933	65.97999	11.78372	11.435 6
4	11.106	MM	0.0656	365.74561	92.94729	63.390 9
5	12.929	MM	0.0825	40.96040	8.27779	7.099 2
Totals :				576.96859	140.67426	

Note: An impurity peak that is seen at 11.1 min in the blank appears in the HPLC traces of all the characterized peptides.

Lys₁₀-teixobactin **1**

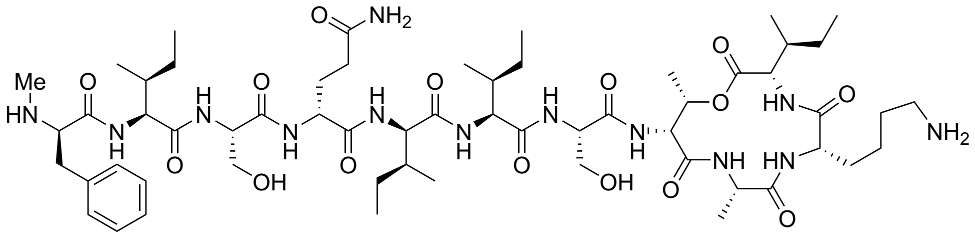

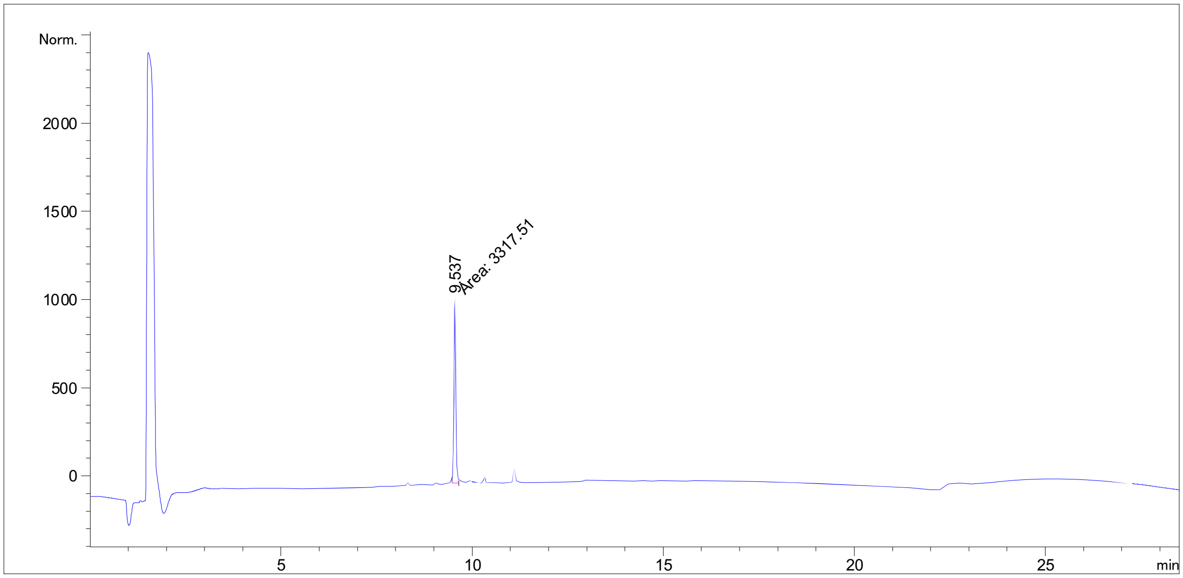


```
Peak RetTime Type  Width     Area      Height     Area
#   [min]        [min]   [mAU*s]     [mAU]         %
----|-------|----|-------|----------|----------|--------|
1   9.537 MM    0.0583 3317.50903  948.15326 100.000   0

Totals :                 3317.50903  948.15326
```

Calculated mass for Lys_{10}-teixobactin: 1215.7227
$[M+H]^+$ = 1216.7300
$[M+2H]^{2+}$ = 608.8689

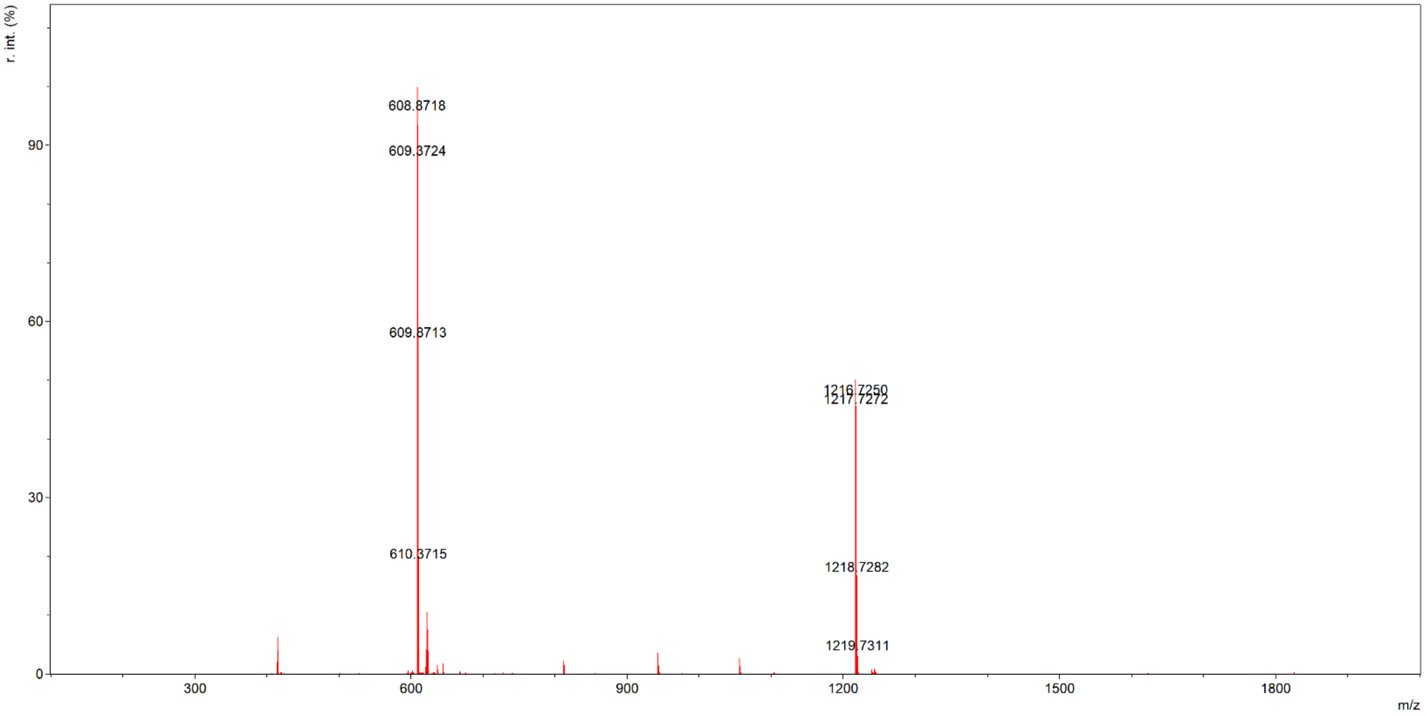

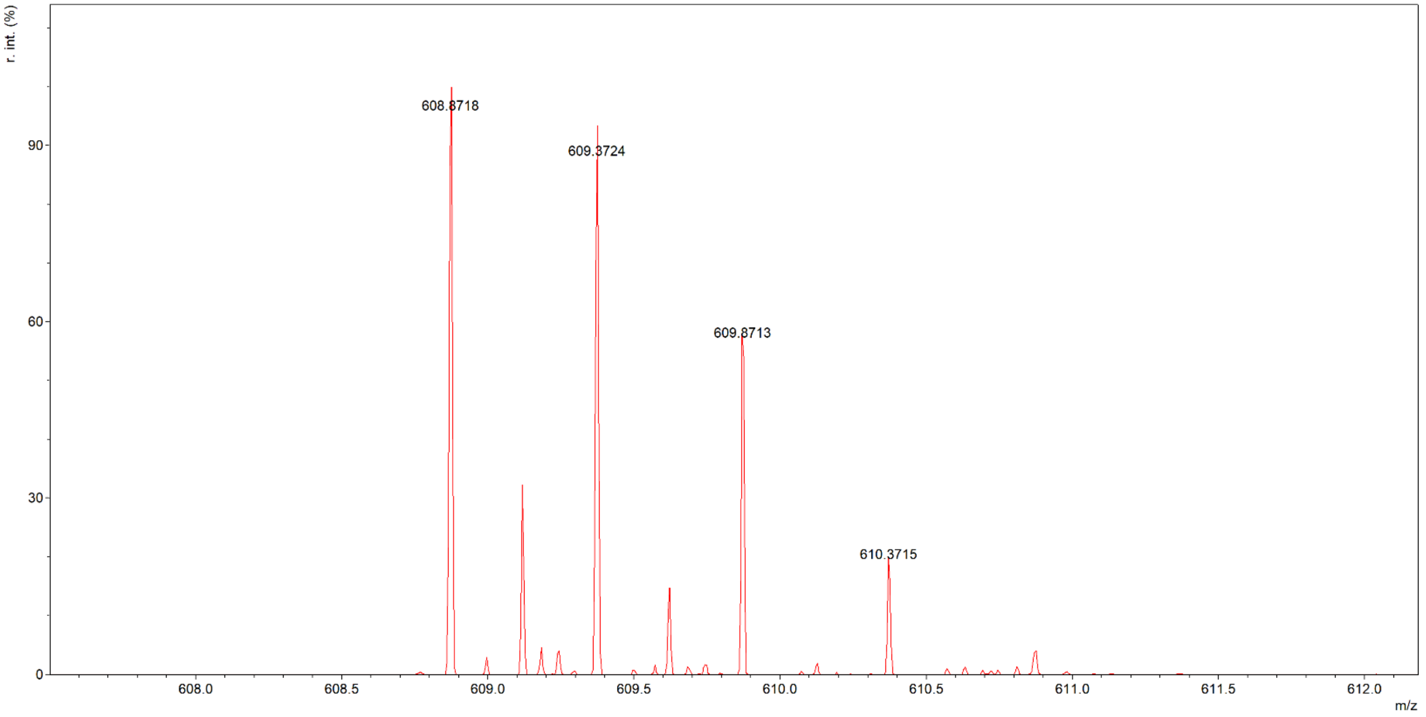

[Ile$_6$-O-Ser$_7$],Lys$_{10}$-teixobactin **2**

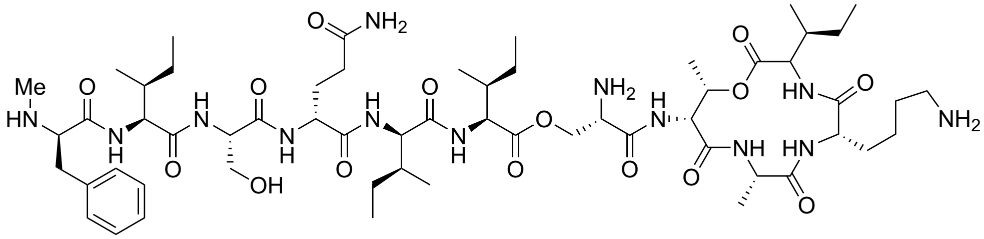

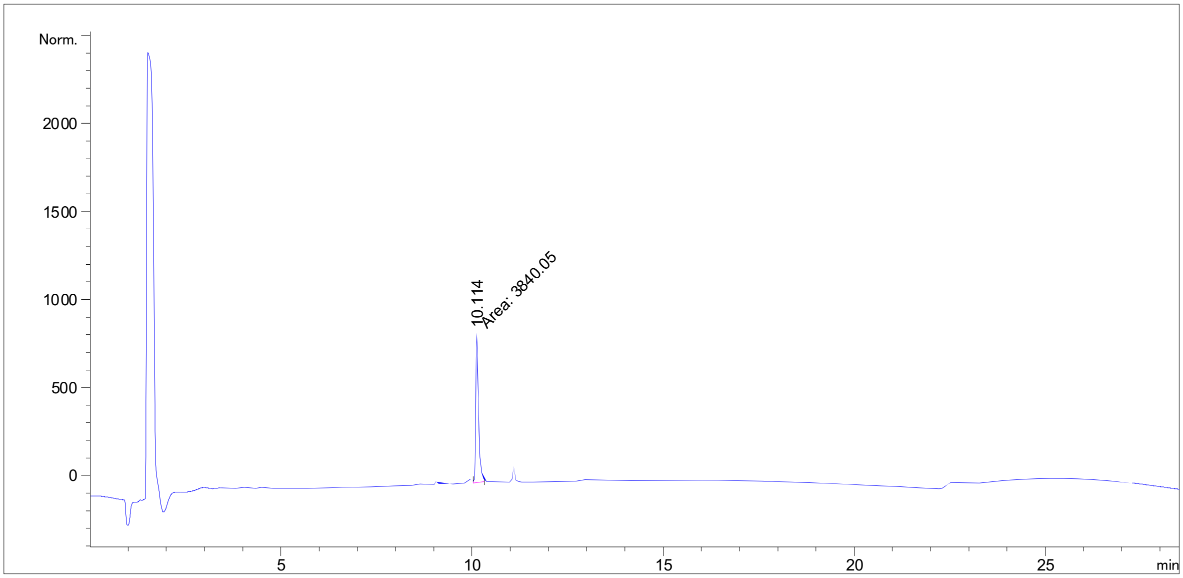

Peak #	RetTime [min]	Type	Width [min]	Area [mAU*s]	Height [mAU]	Area %	
1	10.114	MM	0.0827	3840.05347	773.84924	100.000	0

Totals :	3840.05347	773.84924

Calculated mass for [Ile_6-O-Ser_7],Lys_{10}-teixobactin: 1215.7227
$[M+H]^+$ = 1216.7300
$[M+2H]^{2+}$ = 608.8689
$[M+3H]^{3+}$ = 406.2482

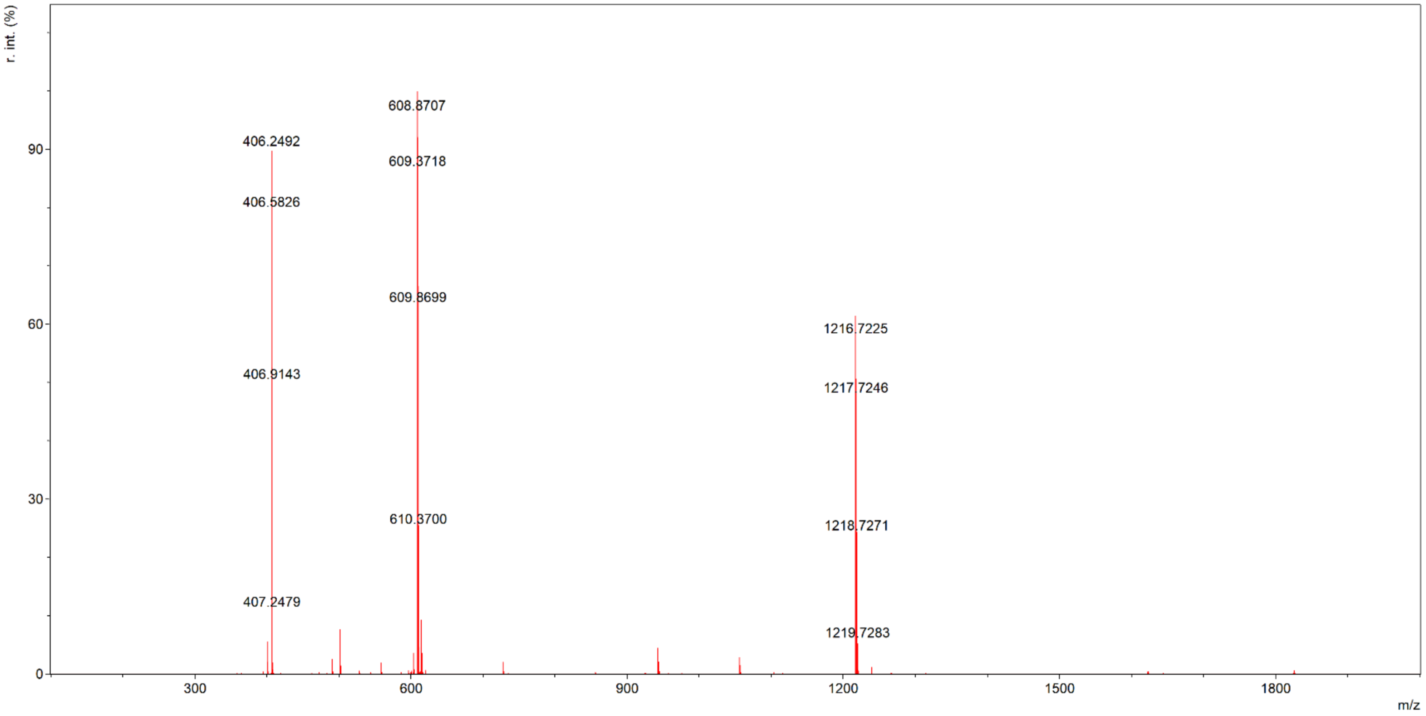

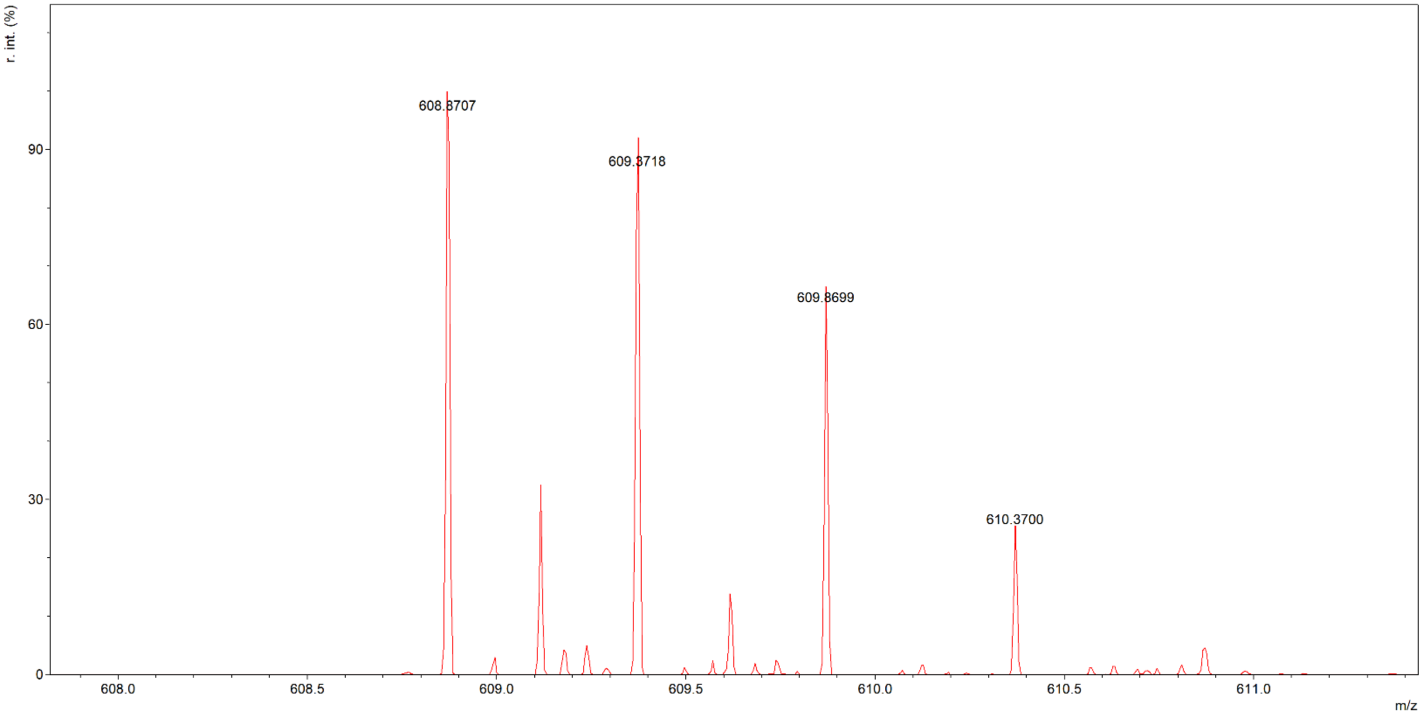

Ile$_4$,Gln$_5$,[Ile$_6$-O-Ser$_7$],Lys$_{10}$-teixobactin ***3***

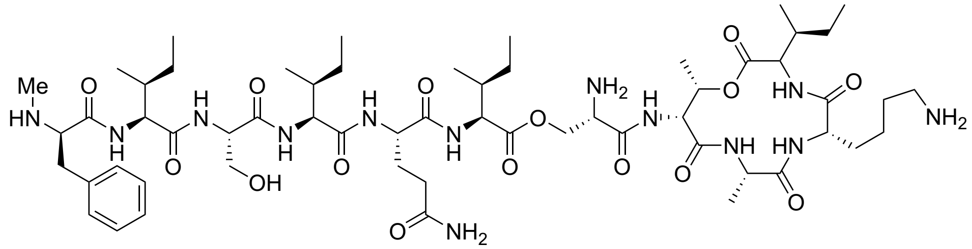

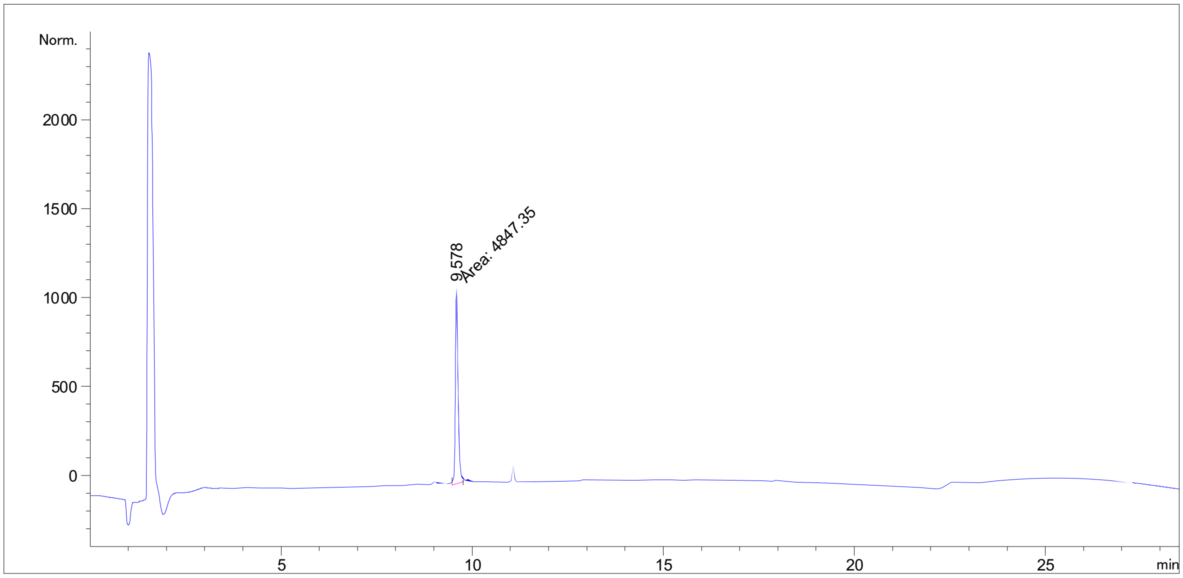

Peak #	RetTime [min]	Type	Width [min]	Area [mAU*s]	Height [mAU]	Area %
1	9.578	MM	0.0806	4847.34863	1002.93707	100.000

Totals : 4847.34863 1002.93707

Calculated mass for Ile_4,Gln_5,[Ile_6-O-Ser_7],Lys_{10}-teixobactin: 1215.7227
$[M+H]^+ = 1216.7300$
$[M+2H]^{2+} = 608.8689$
$[M+3H]^{3+} = 406.2482$

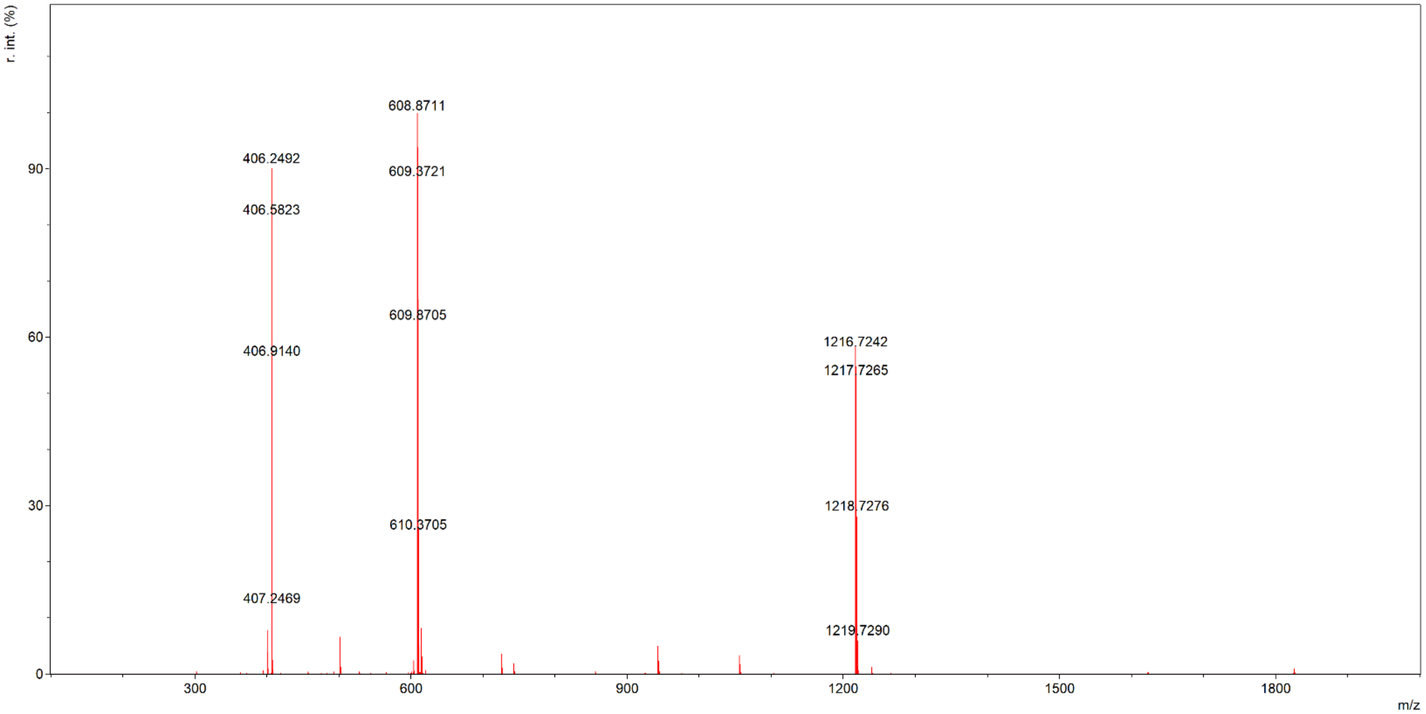

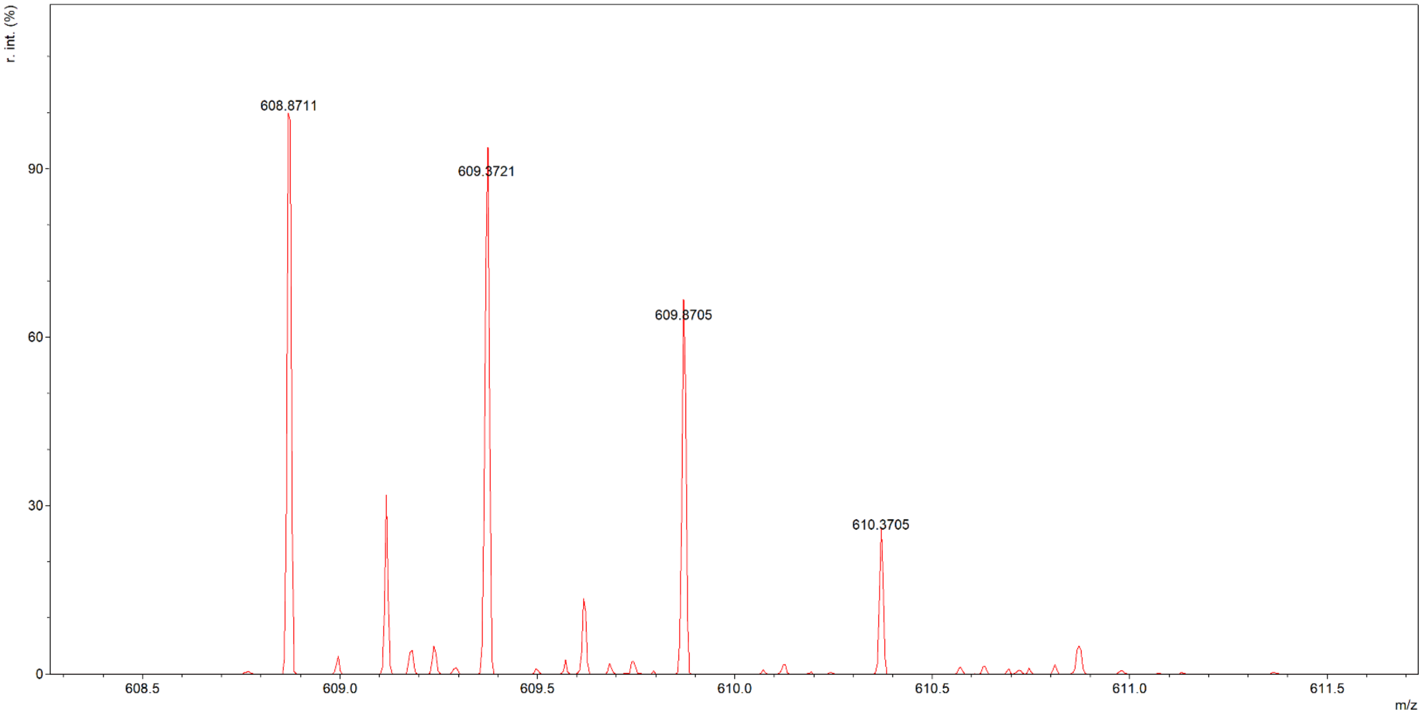

*N-Me-*L*-Phe*$_1$*,Ile*$_4$*,Gln*$_5$*,[Ile*$_6$*-O-Ser*$_7$*],Lys*$_{10}$*-teixobactin* ***4***

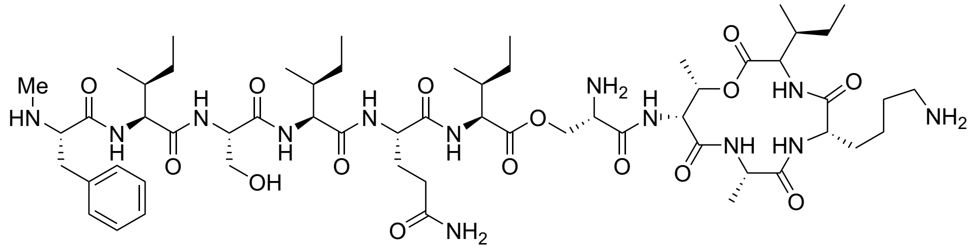

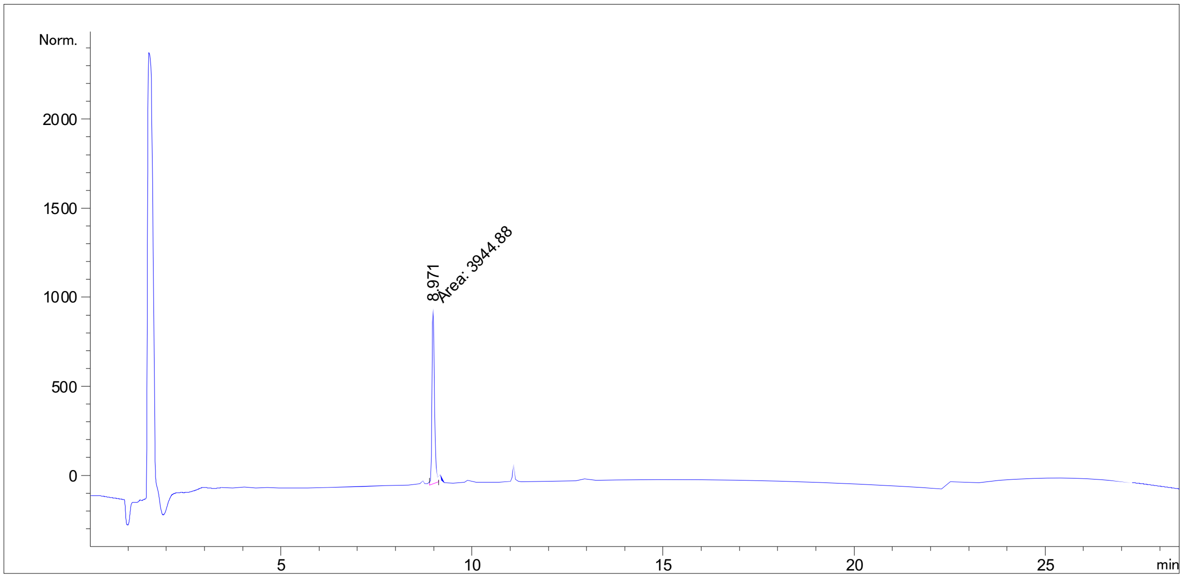

Peak #	RetTime [min]	Type	Width [min]	Area [mAU*s]	Height [mAU]	Area %	
1	8.971	MM	0.0729	3944.88159	902.38306	100.000	0

Totals : 3944.88159 902.38306

Calculated mass for *N*-Me-L-Phe$_1$,Ile$_4$,Gln$_5$,[Ile$_6$-O-Ser$_7$],Lys$_{10}$-teixobactin: 1215.7227
$[M+H]^+$ = 1216.7300
$[M+2H]^{2+}$ = 608.8689
$[M+3H]^{3+}$ = 406.2482

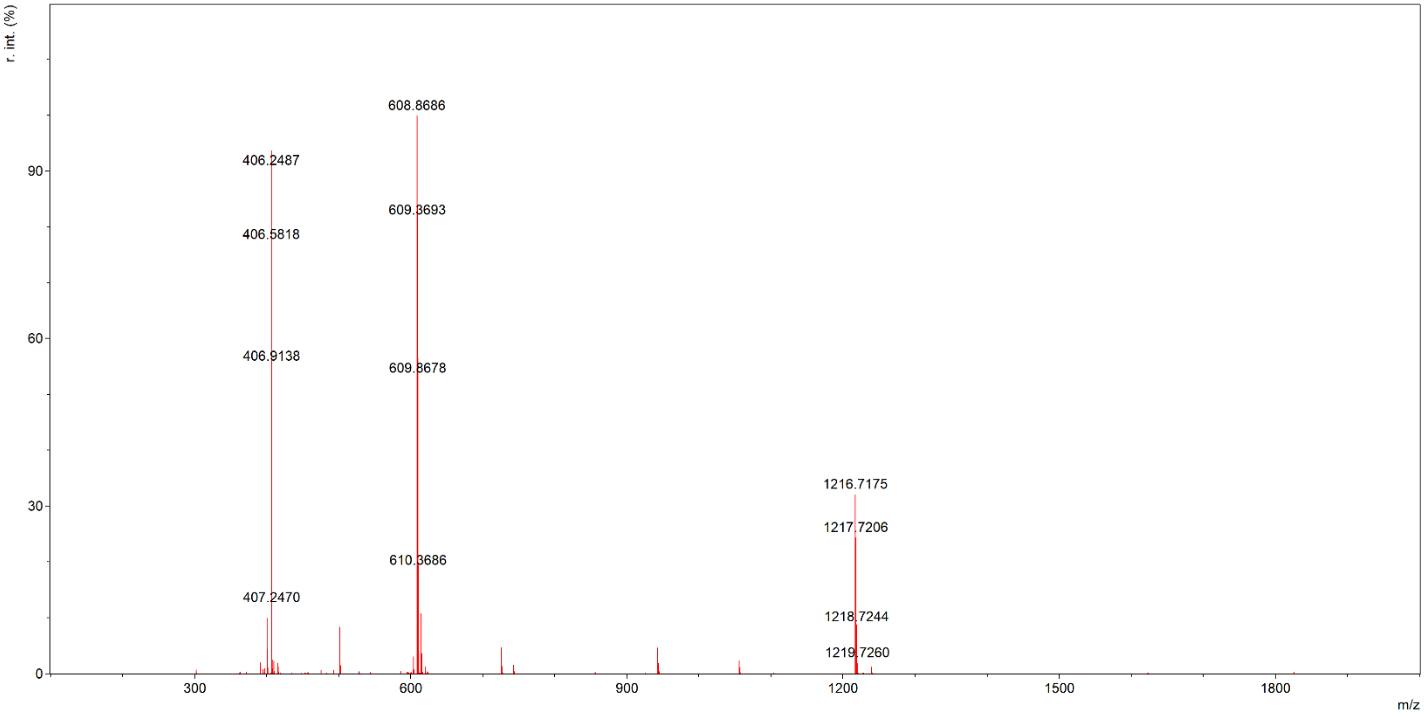

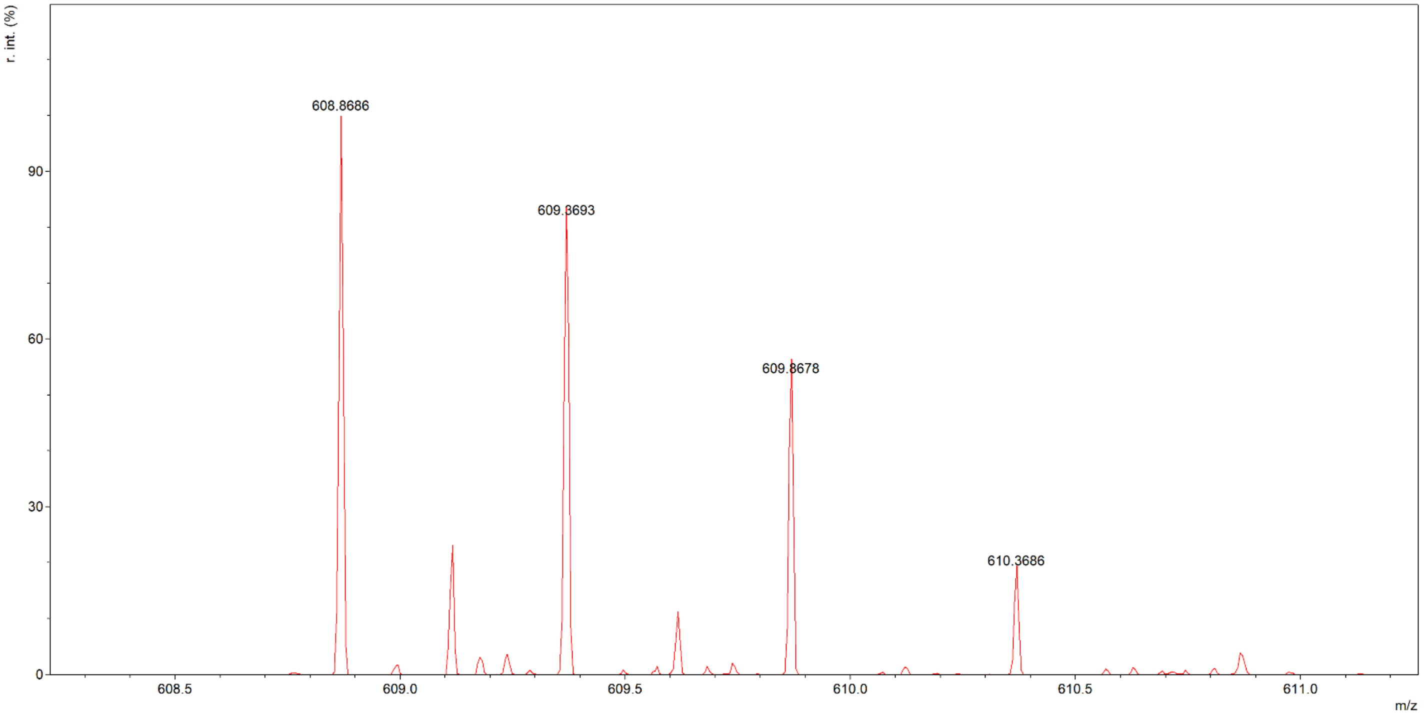

N-Bn-Gly$_1$,Ile$_4$,Gln$_5$,[Ile$_6$-O-Ser$_7$],Lys$_{10}$-teixobactin **5**

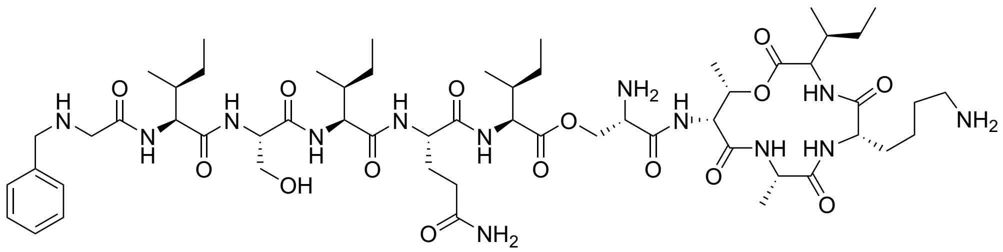

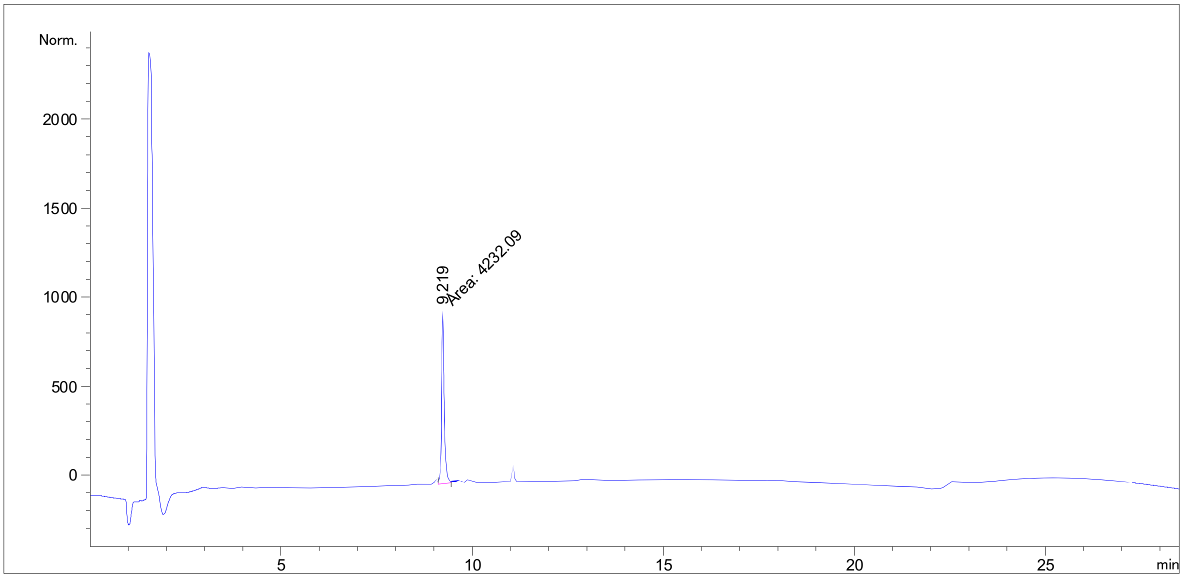

Peak #	RetTime [min]	Type	Width [min]	Area [mAU*s]	Height [mAU]	Area %	
1	9.219	MM	0.0797	4232.09375	884.69745	100.000	0
Totals :				4232.09375	884.69745		

Calculated mass for *N*-Bn-Gly$_1$,Ile$_4$,Gln$_5$,[Ile$_6$-O-Ser$_7$],Lys$_{10}$-teixobactin: 1201.7071
$[M+H]^+$ = 1202.7143
$[M+2H]^{2+}$ = 601.8608
$[M+3H]^{3+}$ = 401.5763

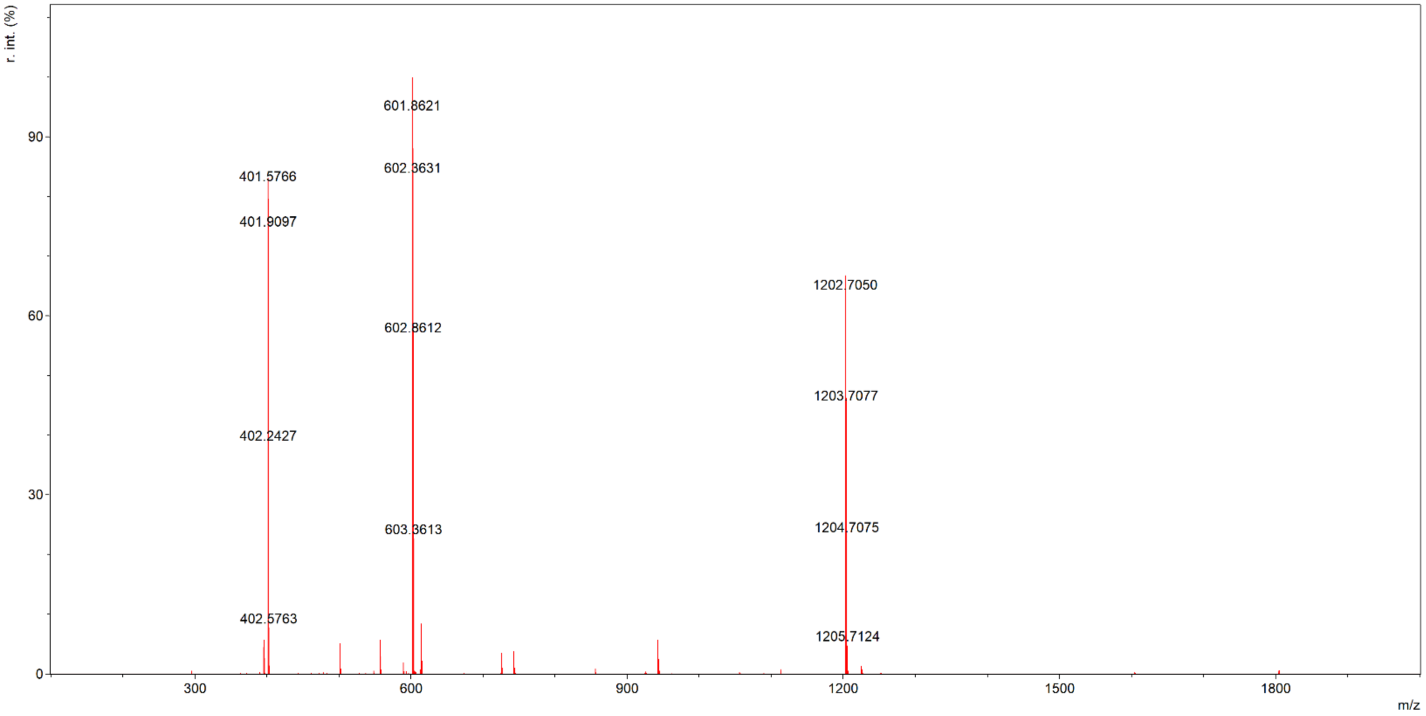

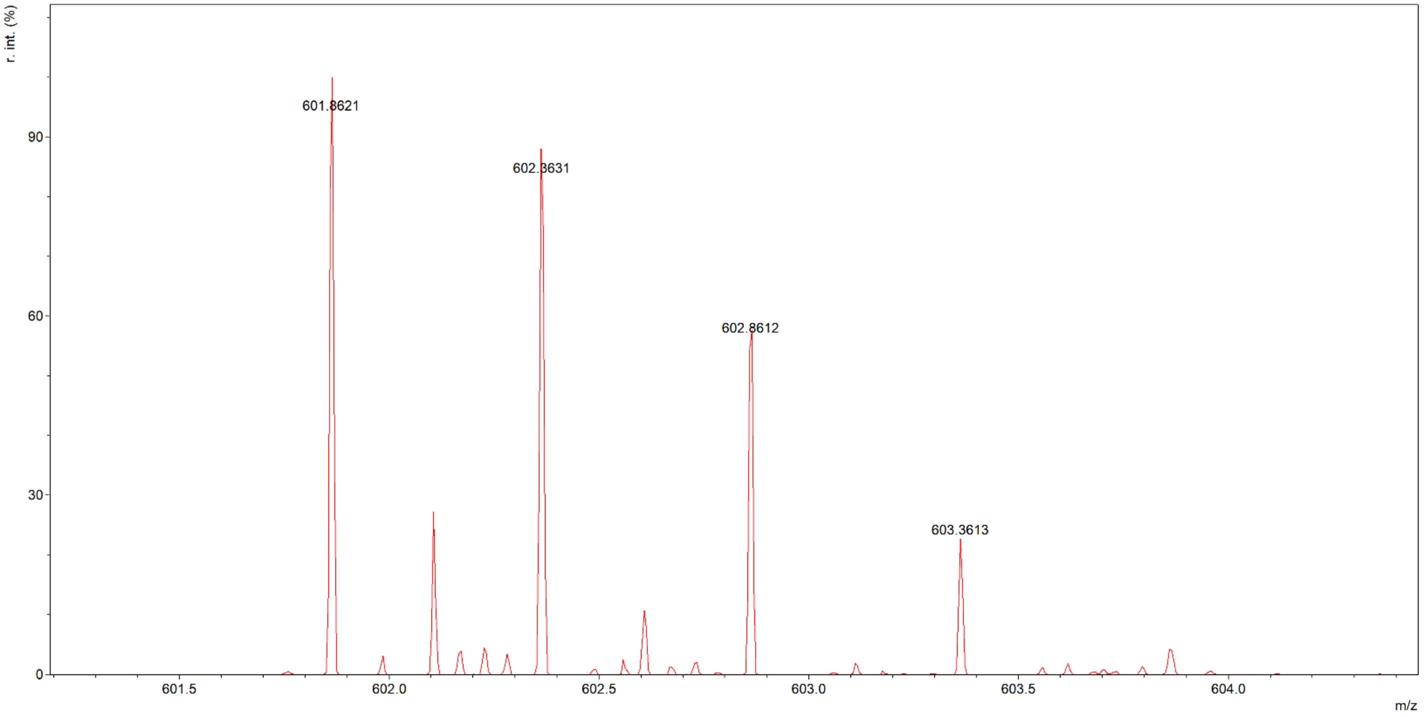

Arg₁₀-teixobactin ***6***

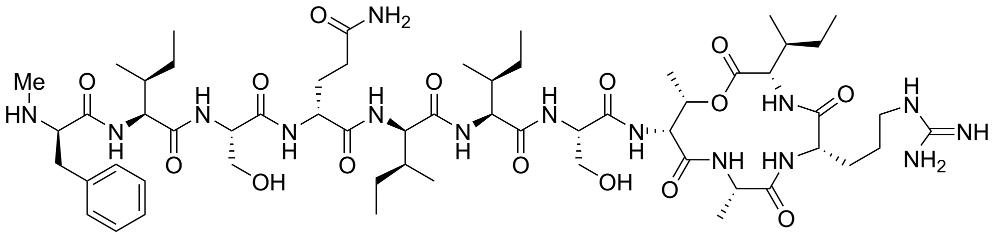

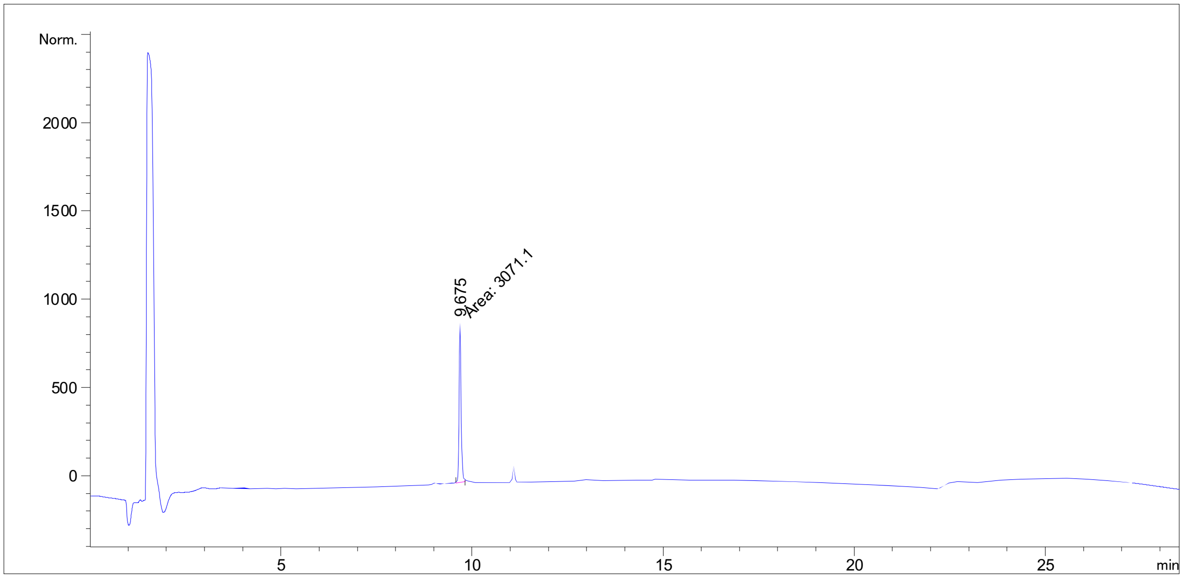

Peak #	RetTime [min]	Type	Width [min]	Area [mAU*s]	Height [mAU]	Area %
1	9.675	MM	0.0622	3071.09692	823.41827	100.000 0

Totals : 3071.09692 823.41827

Calculated mass for Arg_{10}-teixobactin: 1243.7289
$[M+H]^+$ = 1244.7361
$[M+2H]^{2+}$ = 622.8717

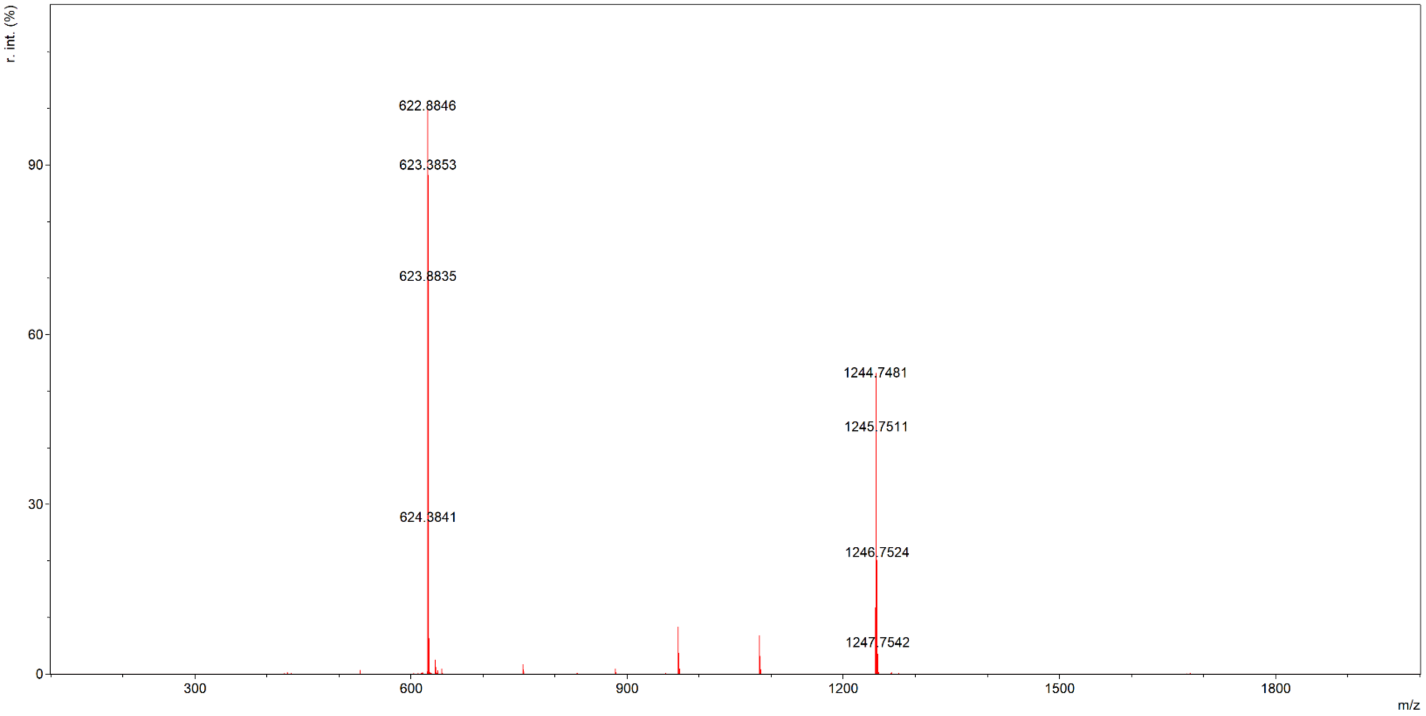

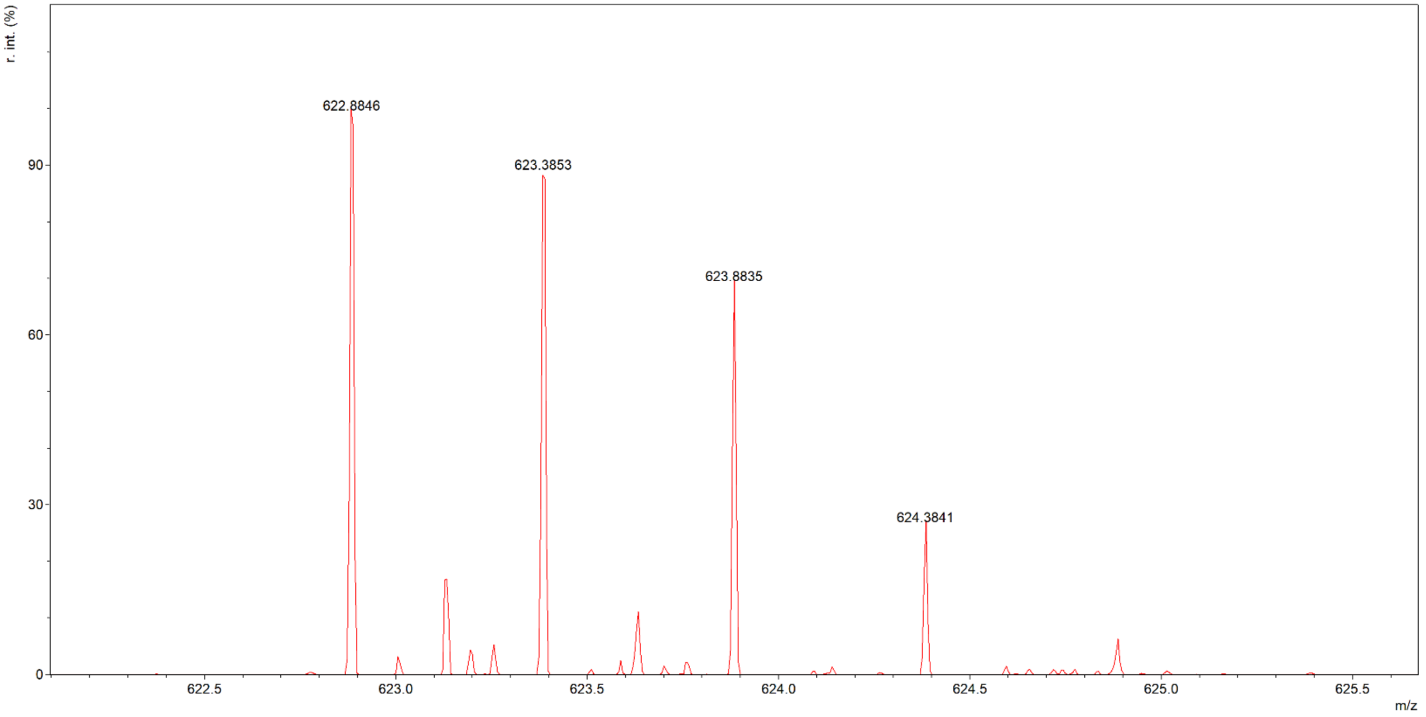

[Ile$_6$-O-Ser$_7$],Arg$_{10}$-teixobactin 7

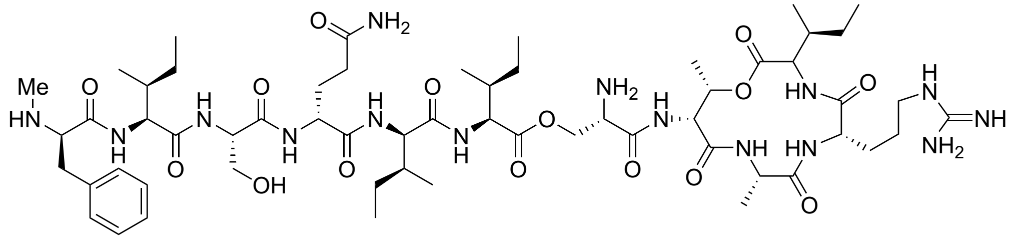

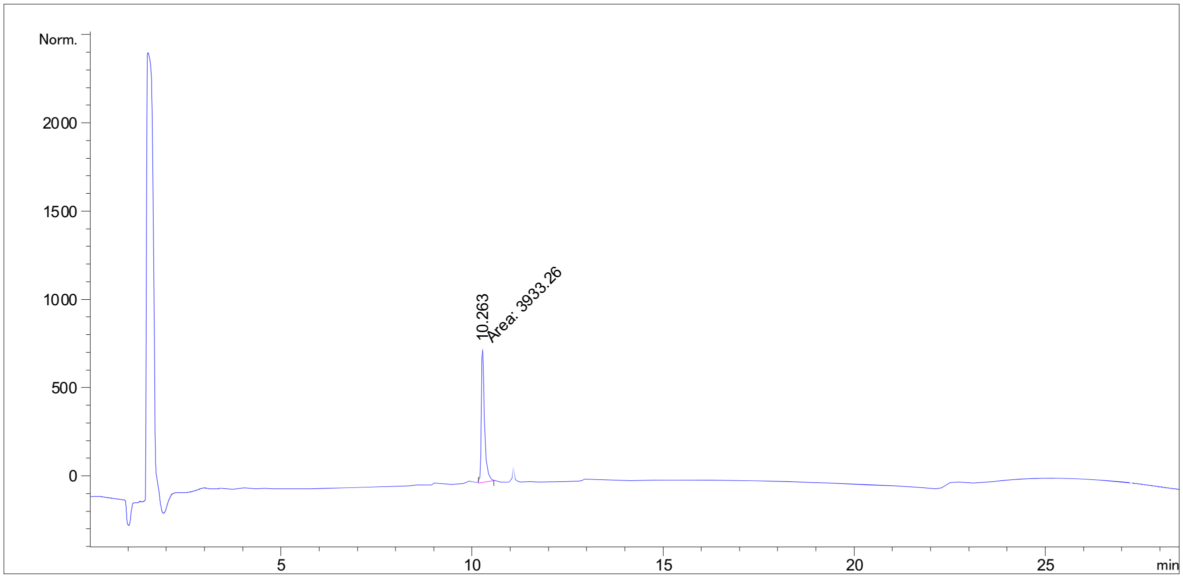

Peak #	RetTime [min]	Type	Width [min]	Area [mAU*s]	Height [mAU]	Area %	
1	10.263	MM	0.0938	3933.26465	698.89679	100.000	0

Totals :		3933.26465	698.89679

Calculated mass for [Ile$_6$-O-Ser$_7$],Arg$_{10}$-teixobactin: 1243.7289
$[M+H]^+$ = 1244.7361
$[M+2H]^{2+}$ = 622.8717
$[M+3H]^{3+}$ = 415.5836

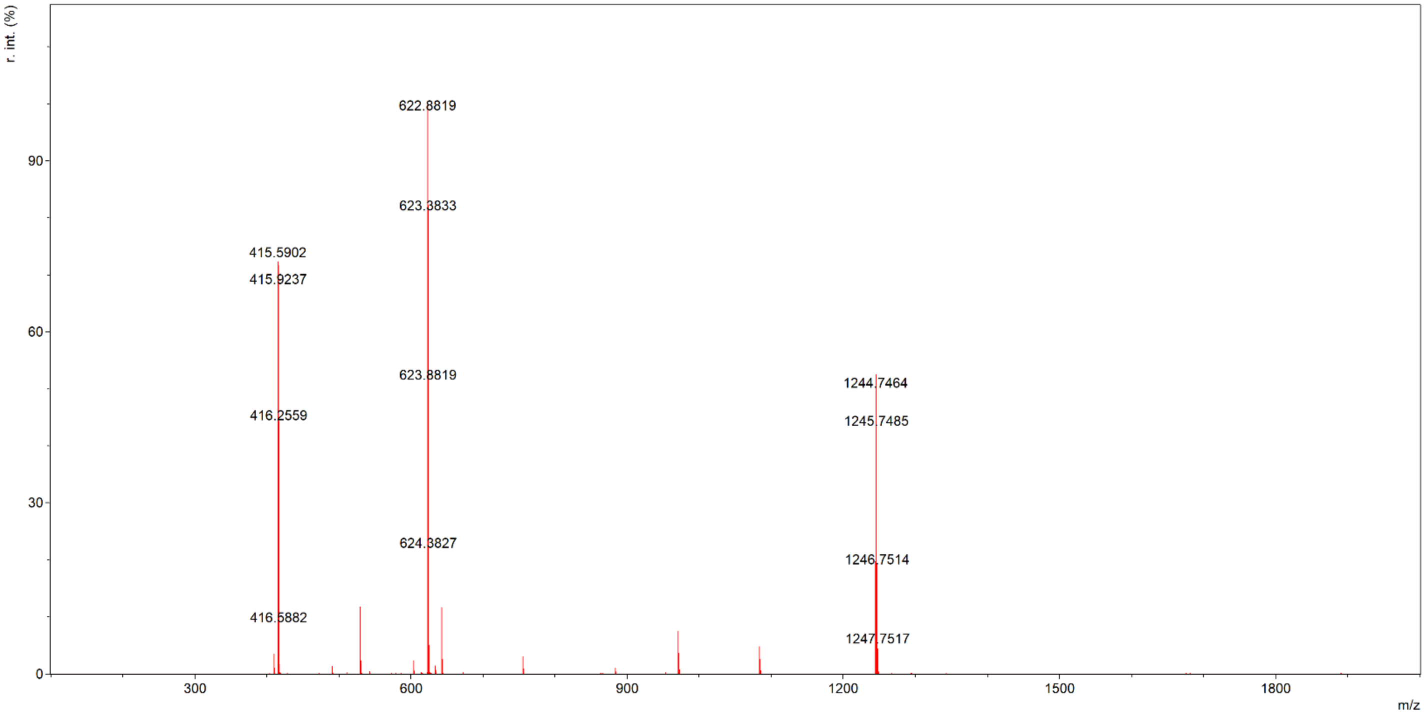

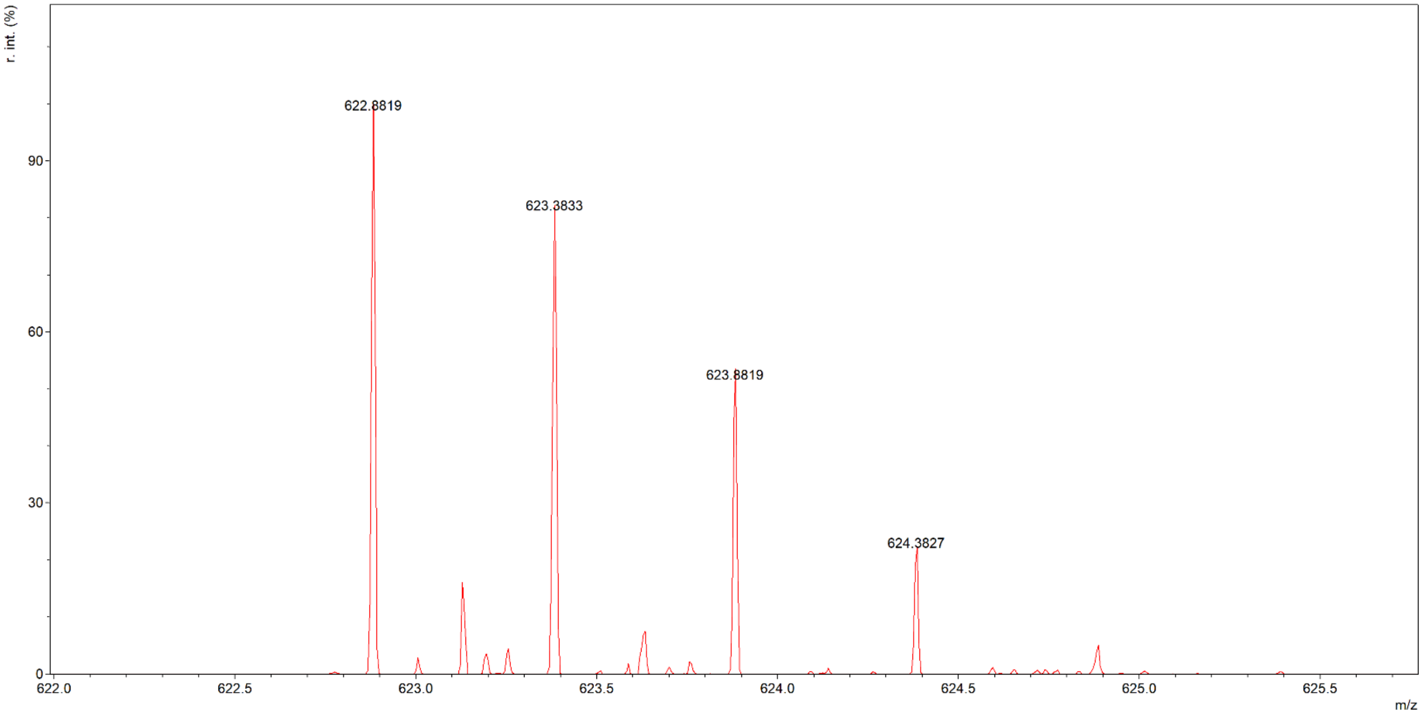

Ile$_4$,Gln$_5$,[Ile$_6$-O-Ser$_7$],Arg$_{10}$-teixobactin ***8***

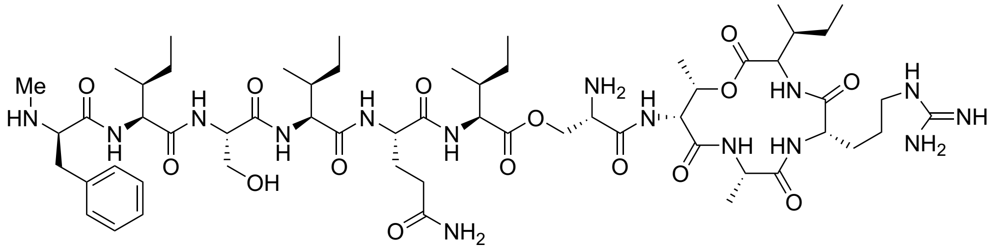

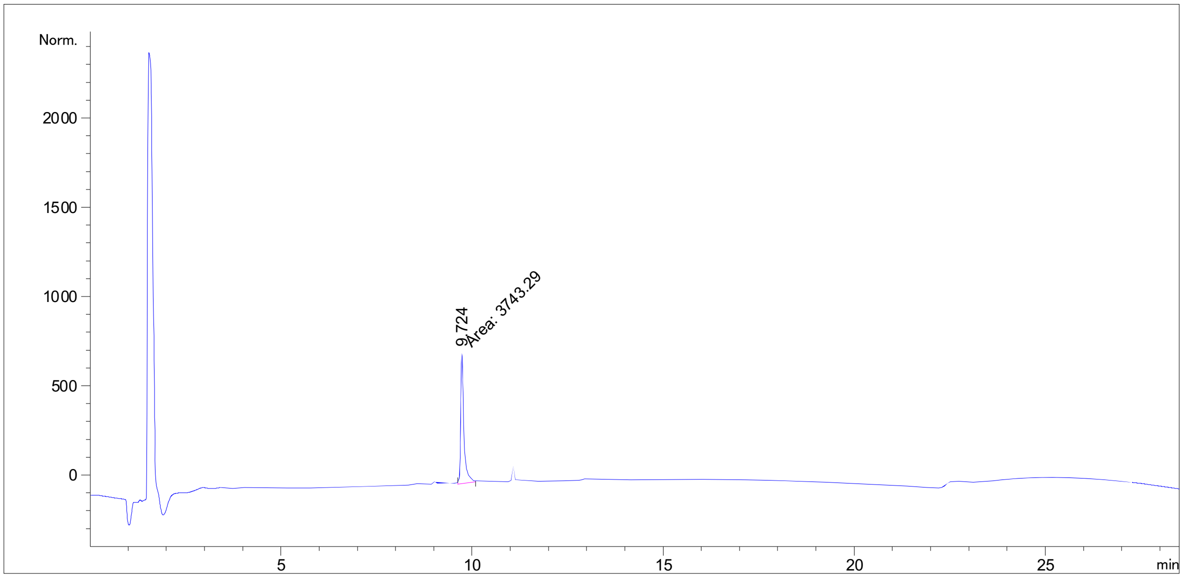

Peak #	RetTime [min]	Type	Width [min]	Area [mAU*s]	Height [mAU]	Area %	
1	9.724	MM	0.0928	3743.28955	672.38824	100.000	0
Totals :				3743.28955	672.38824		

Calculated mass for Ile_4,Gln_5,[Ile_6-O-Ser_7],Arg_{10}-teixobactin: 1243.7289
$[M+H]^+ = 1244.7361$
$[M+2H]^{2+} = 622.8717$
$[M+3H]^{3+} = 415.5836$

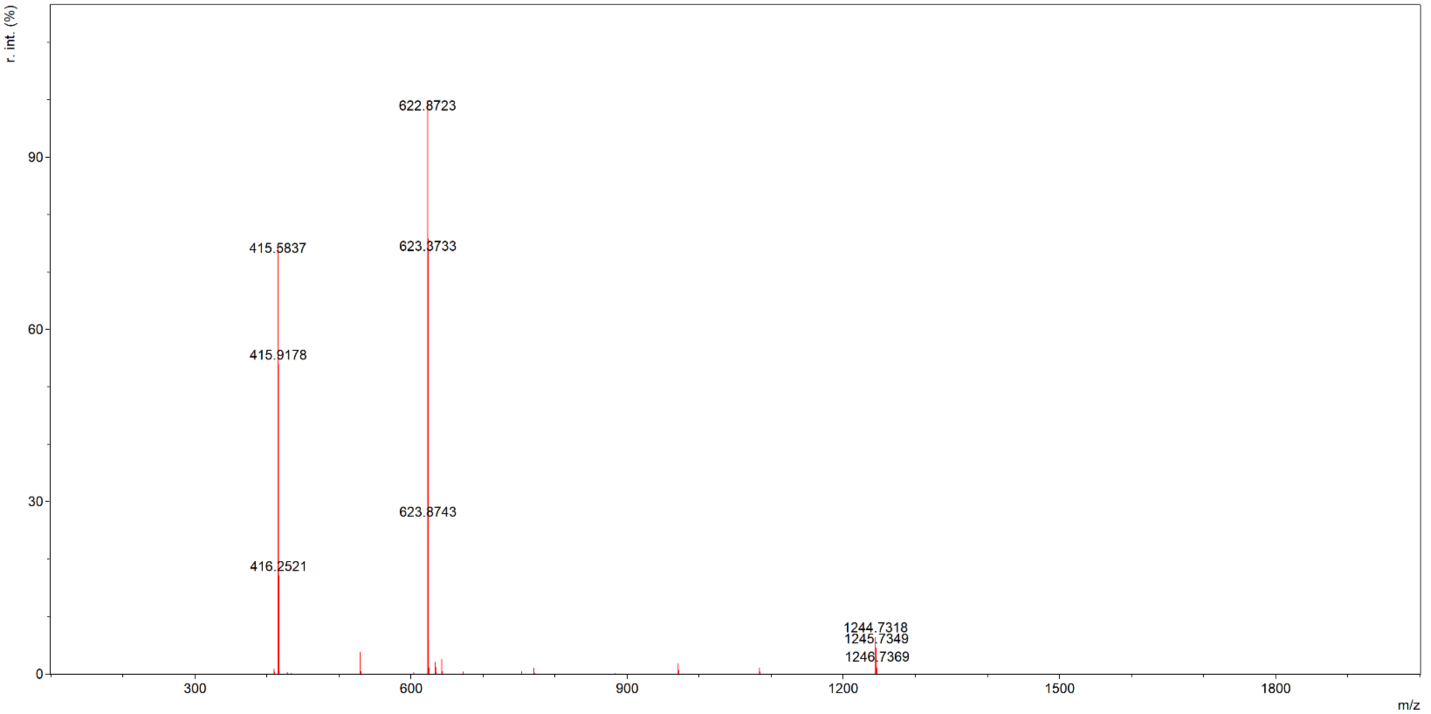

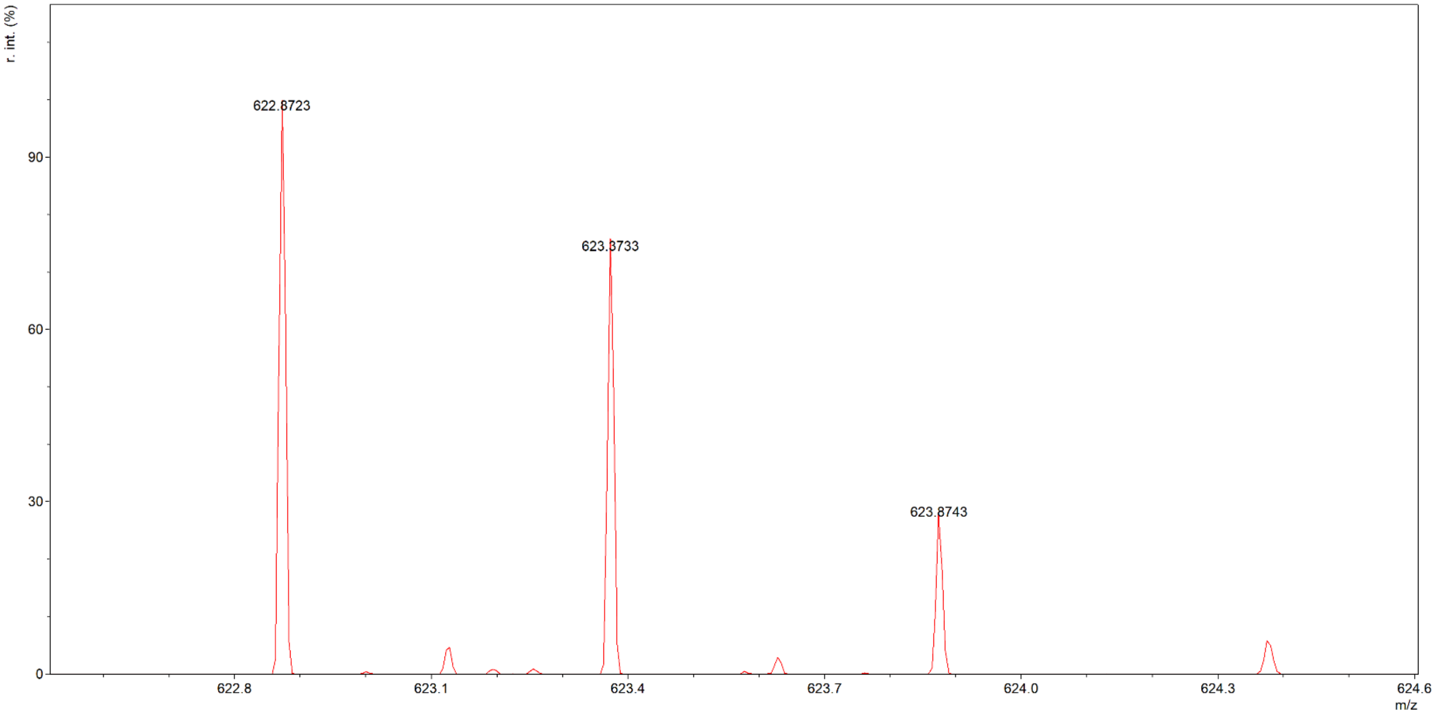

N-Me-Phe$_0$,Gln$_1$,Ile$_4$,Gln$_5$,[Ile$_6$-O-Ser$_7$],Arg$_{10}$-teixobactin ***9***

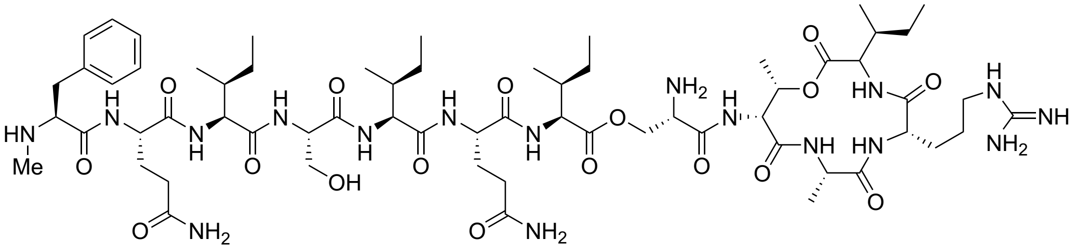

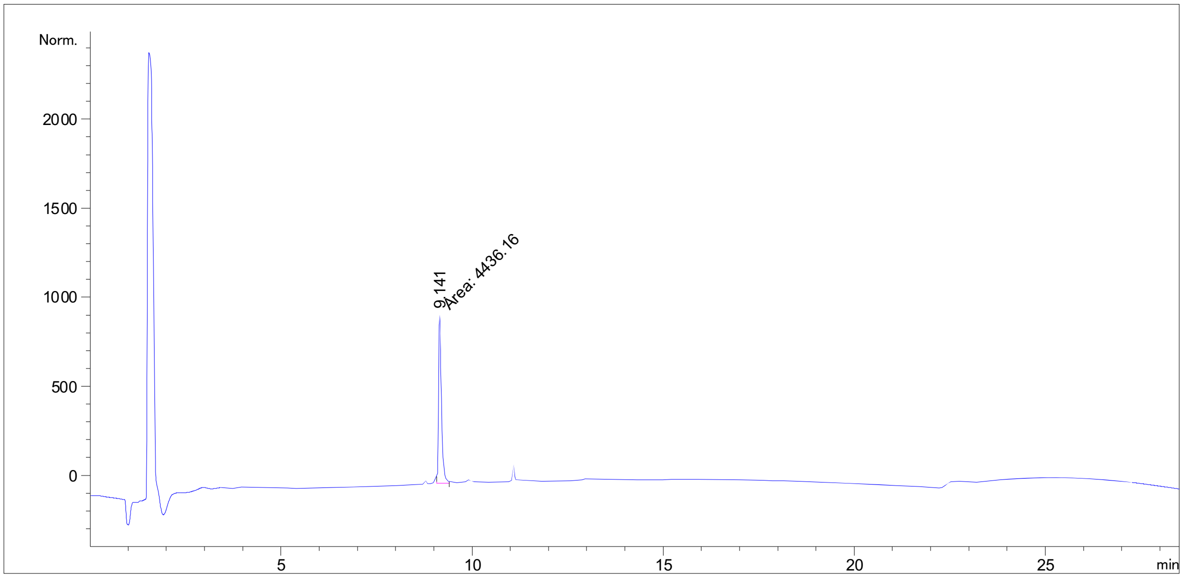

Signal 1: MWD1 A, Sig=214,4 Ref=off

Peak #	RetTime [min]	Type	Width [min]	Area [mAU*s]	Height [mAU]	Area %
1	9.141	MM	0.0858	4436.16162	861.54309	100.000

0

Totals : 4436.16162 861.54309

Calculated mass for *N*-Me-Phe$_0$,Gln$_1$,Ile$_4$,Gln$_5$,[Ile$_6$-O-Ser$_7$],Arg$_{10}$-teixobactin: 1371.7874
$[M+H]^+$ = 1372.7947
$[M+2H]^{2+}$ = 686.9010
$[M+3H]^{3+}$ = 458.2698

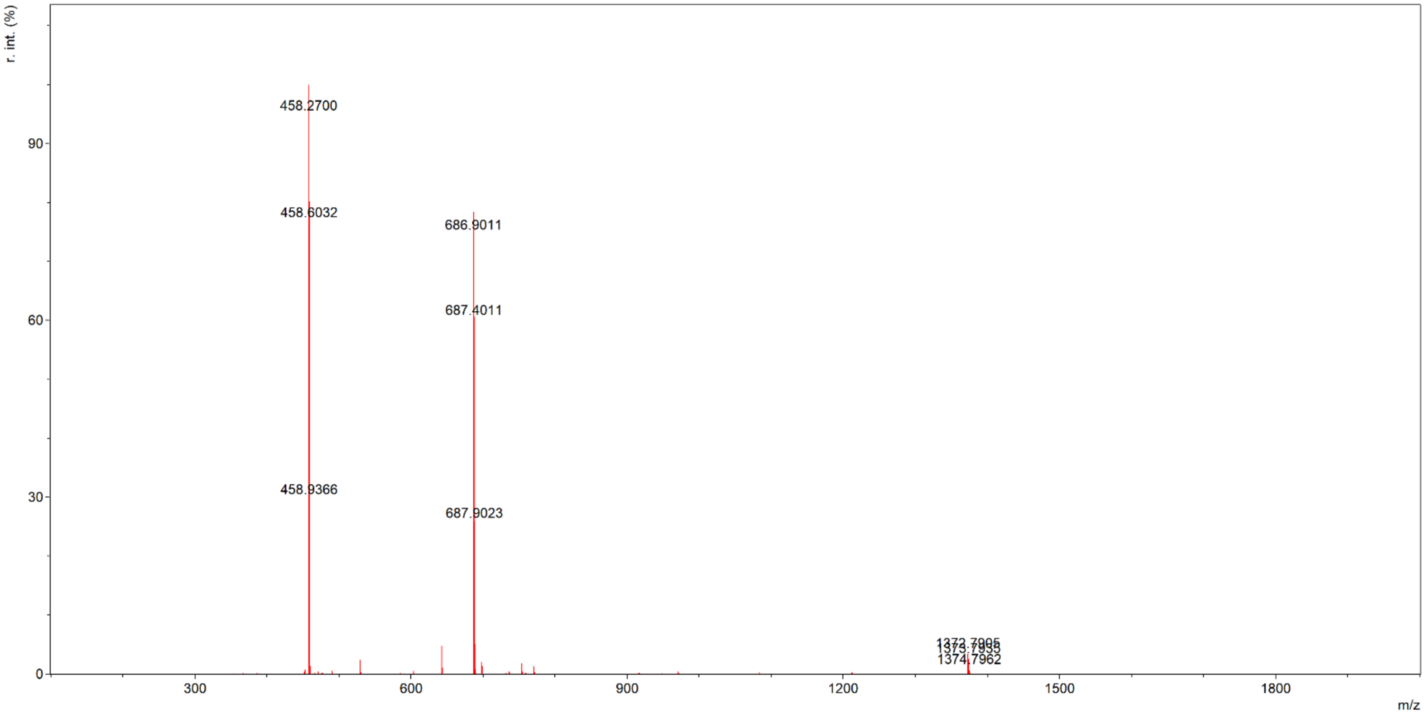

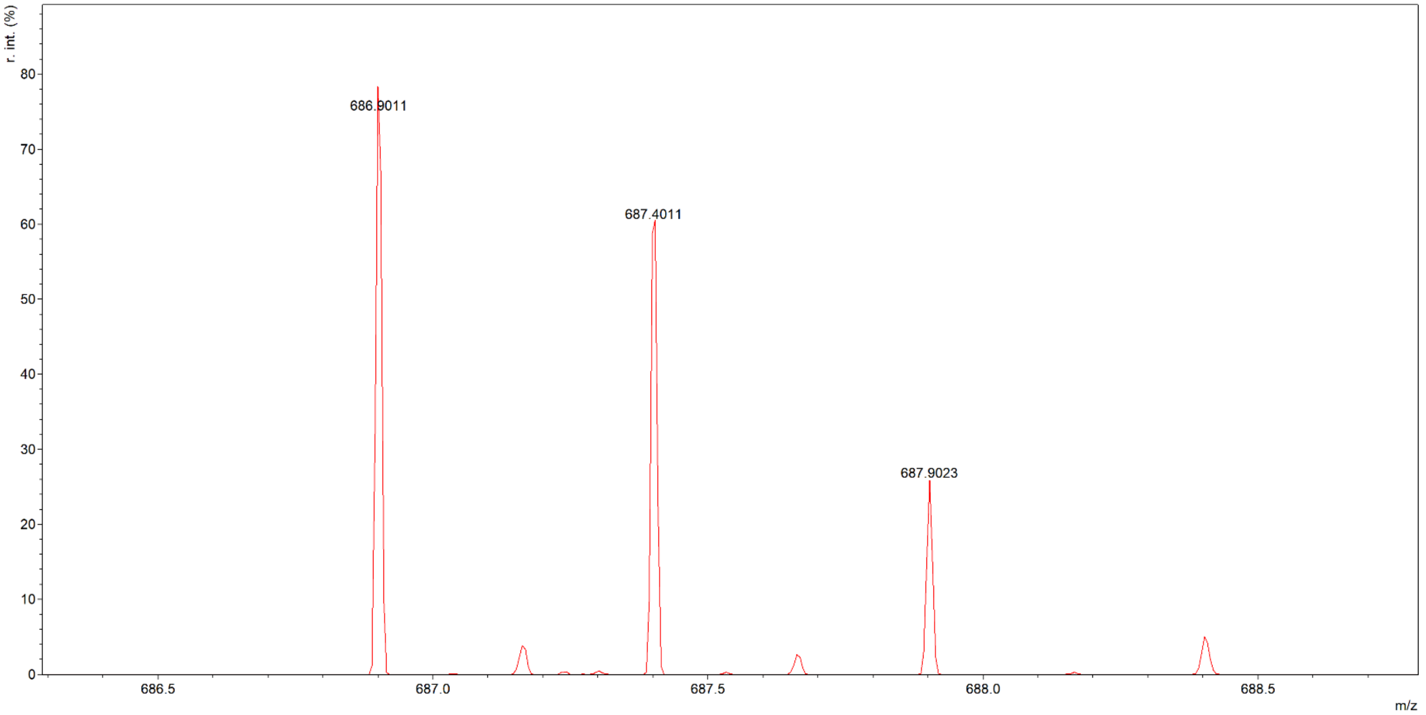

Supplement: Supplementary file 1 — jo4c01674_si_001.pdf [file jo4c01674_si_001.pdf]
